# Investigating the Role of TNF-α and IFN-γ Activation on the Dynamics of iNOS Gene Expression in LPS Stimulated Macrophages

**DOI:** 10.1371/journal.pone.0153289

**Published:** 2016-06-08

**Authors:** Taha Salim, Cheryl L. Sershen, Elebeoba E. May

**Affiliations:** Department of Biomedical Engineering, University of Houston, Houston, Texas, United States of America; University of California Irvine, UNITED STATES

## Abstract

Macrophage produced inducible nitric oxide synthase (iNOS) is known to play a critical role in the proinflammatory response against intracellular pathogens by promoting the generation of bactericidal reactive nitrogen species. Robust and timely production of nitric oxide (NO) by iNOS and analogous production of reactive oxygen species are critical components of an effective immune response. In addition to pathogen associated lipopolysaccharides (LPS), iNOS gene expression is dependent on numerous proinflammatory cytokines in the cellular microenvironment of the macrophage, two of which include interferon gamma (IFN-γ) and tumor necrosis factor alpha (TNF-α). To understand the synergistic effect of IFN-γ and TNF-α activation, and LPS stimulation on iNOS expression dynamics and NO production, we developed a systems biology based mathematical model. Using our model, we investigated the impact of pre-infection cytokine exposure, or priming, on the system. We explored the essentiality of IFN-γ priming to the robustness of initial proinflammatory response with respect to the ability of macrophages to produce reactive species needed for pathogen clearance. Results from our theoretical studies indicated that IFN-γ and subsequent activation of IRF1 are essential in consequential production of iNOS upon LPS stimulation. We showed that IFN-γ priming at low concentrations greatly increases the effector response of macrophages against intracellular pathogens. Ultimately the model demonstrated that although TNF-α contributed towards a more rapid response time, measured as time to reach maximum iNOS production, IFN-γ stimulation was significantly more significant in terms of the maximum expression of iNOS and the concentration of NO produced.

## Introduction

The inducible nitric oxide synthase (iNOS) enzyme plays a critical role in the primary proinflammatory response in macrophages upon pathogen infection. Inhibition and mutation of iNOS have resulted in diminished immunological response against intracellular pathogens [1, 2]. Therefore, understanding the mechanism of the iNOS gene expression system and the dynamics of nitric oxide (NO) production upon exposure to lipopolysaccharides (LPS) and inflammatory cytokines can provide additional insight regarding the initiation of the macrophage effector response. Empirically, in addition to LPS and cytokine bioavailability, cytokine synergism as well as antagonism (i.e., crosstalk) is known to be highly consequential in immune response [3–9]. However, few studies have successfully quantified the mechanistic contribution of cytokine synergism to the temporal dynamics of immune effector response. Given the centrality of effector molecules like nitric oxide in the containment and eventual clearance of pathogenic infections, successfully correlating the host cytokine environment to immunological response can aid in the development of targeted immunomodulatory therapies to combat infection.

An effective tool for understanding the dynamics of complex biological systems is *in silico* modeling. Theoretical models are useful for aggregating empirical observations into quantitative descriptions of the temporal outcome of biochemical interactions in signal transduction cascades and gene expression systems [10, 11]. For the present study, we developed an integrated computational model of the macrophage proinflammatory response to infection and consequential activation of iNOS gene expression. Using our model, we investigated the contribution of the local cytokine environment to the actuation of the effector response by exploring the synergy between tumor necrosis factor alpha (TNF-α) and interferon gamma (IFN-γ) and the consequences of this synergy on the dynamics of iNOS production.

## Background

Macrophages are phagocytic cells that are able to recognize a wide array of signaling molecules such as immune stimulatory lipopolysaccharides (endotoxin or LPS) shed from the cell walls of gram-negative bacteria, and cytokines, including IFN-γ and TNF-α, produced by other immune cells. The binding of these signaling factors to their corresponding receptors initiates a signal transduction cascade that induces the macrophage proinflammatory response, leading to the production of additional cytokines, chemokines, and the activation of proinflammatory genes. The expression of proinflammatory genes such as iNOS and the concomitant effector proteins leads to the production of reactive oxygen species (ROS) and reactive nitrogen species that directly antagonize intracellular pathogens [12, 13].

Two major pathways are known to influence iNOS gene expression within macrophages, the mitogen-activated protein kinase (MAPK) pathway and the Janus kinase (JAK) and Signal Transducer and Activator of Transcription (STAT) pathway, collectively known as the JAK/STAT pathway. LPS and TNF-α activate the MAPK pathway, which proceeds to activate nuclear factor kappa-B (NF-κB) and activator protein-1 (AP1), key proinflammatory transcription factors [14, 15]. IFN-γ on the other hand, activates the JAK/STAT pathway [16]. Activation of the JAK/STAT pathway leads to the formation of phosphorylated STAT1 dimers and activation of the interferon regulatory factor-1 (IRF1), two other key transcription factors used in the regulation of iNOS gene production [17]. Both of these pathways are able to individually induce iNOS expression but the synergy between these pathways greatly amplifies the response [18]. Accordingly, optimal iNOS gene expression is observed in the presence of all four-transcription factors.

Previous kinetic models have had a singular focus, investigating either the impact of the JAK/STAT or MAPK pathways on the activation of proinflammatory gene expression [16, 19]. These models did not consider the synergism between the pathways nor the contribution of the macrophage’s microenvironment to the activation of these pathways and subsequent activation of the NO effector function. Specifically, Yamada et al modeled the dynamics of IFN-γ activation of the JAK/STAT pathway [16]. With their model, they reproduced the temporal expression of the SOCS1 inhibitory protein and its regulatory effects on the activation of STAT1 dimers. Based on *in silico* studies, Yamada et al concluded that the regulatory enzyme, nuclear phosphatase, was the key regulatory factor in the pathway. However, their model did not consider the synergy of other pathways activated by IFN-γ nor was it in the context of intracellular immuno-modulation. In addition to JAK/STAT models, there have been several dynamic models of the MAPK pathway [20, 21]. Gutierrez et al published an *in silico* model of LPS-modulated TLR4-mediated signaling and ultimate activation of the MAPK pathway and proinflammatory genes. They focused on the development of a novel approach to fully describe the dynamics of complex biochemical reactions using canonical reaction mechanisms [20]. However, their model was not used to investigate the dynamic modulation of the MAPK pathway and resulting changes in gene expression based on variations of the host cytokine environment. Huang and Ferrell used their computational model to show that the MAPK pathway can behave like a highly cooperative enzyme that can be modeled using Hill-Equation kinetics [21]. Although their model helped in elucidating the mechanistic behavior of the pathway, it did not contain the upper MAPK pathway nor did it contain regulatory components such as MAPK phosphatases that down regulate the pathway. In addition to these existing models, given the centrality of MAPK pathways in the activation of NF-κB, many models have been developed to investigate the dynamics of NF-κB activation and subsequent nuclear translocation and activation of effector genes by NF-κB [11, 22–24]. While these dynamic models described key signaling pathways that are relevant in the expression of iNOS, the majority of these models do not consider: the synergism between the MAPK and JAK/STAT pathways, the mechanistic impact of the synergy on iNOS activation, nor the contribution of the macrophage’s microenvironment to MAPK-JAK/STAT dynamics and concomitant actuation of the NO effector function. The model presented here integrates and expands upon key components from existing models to capture the synergy between the JAK/STAT and LPS-activated MAPK pathways, thereby creating a more comprehensive representation of LPS and cytokine modulation of iNOS production.

### The Combined Role of TNF-α and IFN-γ on NO Production

In order to mimic *in vivo* modulatory mechanisms of macrophage activation, we considered the relative cytokine concentrations in the host microenvironment that results during the course of the proinflammatory response. Namely, we focused on modeling how varying concentrations of TNF-α and IFN-γ impact the dynamic response of macrophages and their production of effector molecules. Although TNF-α activates inflammatory pathways in infected macrophages and cells in the vicinity of the infected cell by either an autocrine or paracrine mechanism [25]. IFN-γ is known to be a more potent activator of iNOS and subsequent NO production [26, 27]. *In vitro* studies have shown that both, human and murine bone marrow derived macrophages have the ability to secrete IFN-γ but only under the co-activation of IL-12 and IL-18 [28, 29]. We presume that during initial infection, which we represent as LPS exposure in our model, levels of IL-12 and IL-18 are negligible and insufficient to stimulate the production of appreciable levels of IFN-γ by macrophages. Therefore, we exclude the production of IFN-γ by macrophages from our model. However, as natural killer (NK) cells migrate towards infected cells in response to chemokines produced by infected macrophages, NK cells upregulate their production of inflammatory cytokines like IFN-γ resulting in an increase in proinflammatory cytokines in the microenvironment. Given that lymphocytes are one of the main producers of IFN-γ, we posit that the low levels of iNOS expression and NO concentration observed during the initial phase of infection is due in part to the time delay associated with the recruitment of natural killer cells to the site of infection [29–31].

**Fig 1:**
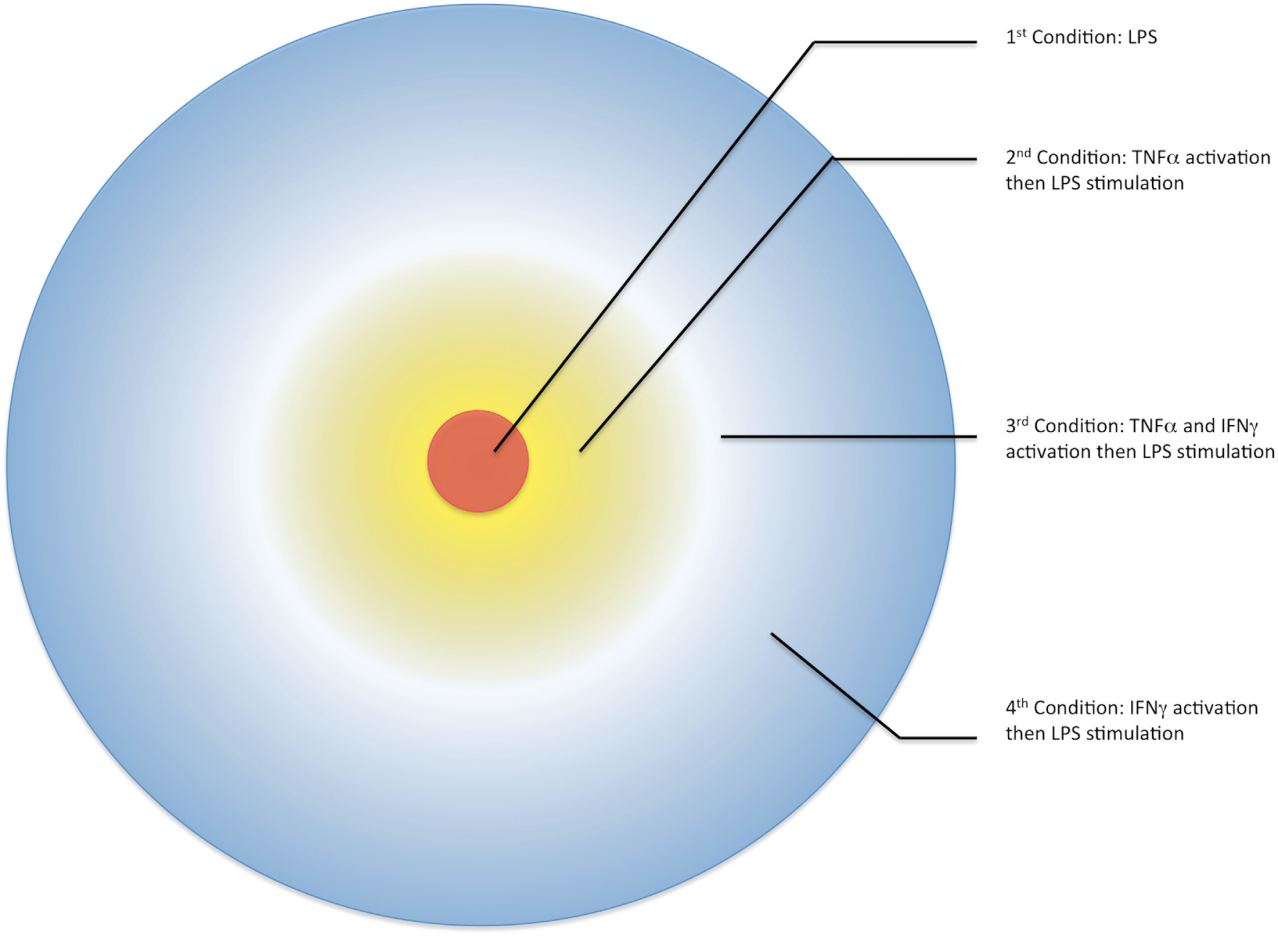
Cytokine Gradient around the Initial Site of Infection in Macrophages. The cytokine gradient or field around the primary infected cell (red) coordinates with the mechanism of the proinflammatory response. As the initially infected macrophage secretes TNF-α (yellow), the TNF-α gradient is highest around the initial cell and radiates outward whereas the IFN-γ (blue) field, which is primarily secreted by peripheral NK cells radiates towards the site of infection. These cytokine fields result in three pre-stimulatory conditions from which cells exhibit dynamically unique proinflammatory responses upon LPS stimulation [30, 35].

As the concentrations of TNF-α and IFN-γ increase in the microenvironment around the infected macrophage, we plausibly speculate that there is an emergence of a cytokine field or a cytokine gradient consisting of varying levels of TNF-α and IFN-γ. Since macrophages at or near the site of infection are activated to produce higher levels of TNF-α and NK cells migrating towards the site of infection are the primary producers of IFN-γ, we propose that during early innate immune response a TNF-α gradient forms with the highest concentration proximal to the initially infected macrophage. Secondly, as a consequence of infiltrating NK cells, we expect the formation of an IFN-γ gradient with the highest concentration at the periphery and decreasing with increasing proximity towards the site of infection (Fig 1). Presumably non-infected macrophages within these cytokine fields are pre-exposed to one or both TNF-α and IFN-γ prior to encountering pathogen-associated LPS. The pre-exposure period is referred to as priming, and cells primed with inflammatory cytokines have been associated with a greater proinflammatory response upon pathogen exposure [26, 27, 32]. The priming condition modulates the macrophage’s ability to more effectively respond and produce bactericidal effector molecules upon infection. Increased pro-inflammatory cytokines help promote a more effective NO and ROS burst in infected macrophages, and *in vivo* and *in vitro* experiments have shown that IFN-γ priming is essential for pathogen clearance in murine macrophages [33, 34].

We hypothesize that differences in the robustness of the initial proinflammatory response between non-primed resting macrophages, which are primary sites of infection, versus macrophages located in the vicinity of infected cells or infiltrating lymphocytes, are directly correlated with the priming of neighboring macrophages and their pre-exposure to TNF-α or/and IFN-γ. We use our *in silico* model to investigate how and to what degree the intracellular mechanism involved in iNOS expression and NO production responds to individual or synergistic IFN-γ and TNF-α priming. We tested our hypothesis by simulating four different activation and priming conditions, where initial values of the stimulatory molecules were greater than zero: LPS alone, LPS with TNF-α, LPS with IFN-γ, and LPS with TNF-α and IFN-γ. Understanding the dynamics of infection-mediated iNOS production in the presence of TNF-α, IFN-γ, and the synergistic effects of both can aid in elucidating each cytokine’s contribution to NO formation and the effector response of the host.

## Model Development and Methods

We develop an integrated model to simulate LPS-mediated and cytokine modulated iNOS activation and NO production. We use a deterministic approach and represent the concentration dynamics of biochemical species as a system of ordinary differential rate equations. Although accounting for stochasticity of molecular interactions when modeling relatively low abundant molecules, such as signaling molecules and genes, has potential benefits, the deterministic modeling approach reduces the computational time for model simulation which allows us to integrate variations in the macrophage’s environment with signal transduction, gene expression, and metabolic outcomes [36]. As in comparable models, the rate equations for our model were derived using both mass action and Michaelis-Menton-based rapid equilibrium kinetics to capture interactions in the signal transduction cascade and production of gene or metabolic substrates, respectively [37–39]. While the aggregate impact of cooperativity among substrates in a reaction cascade, such as components of phospho-relay cascades, can be captured using other kinetic forms like Hill kinetics, we approximate the effective impact of cooperativity by directly modeling the individual binding reactions in the cascade using mass action kinetics [39, 40].

The majority of rate constants were curated from literature and empirically derived while others were obtained from previously published models [41]. The rate constants for all rate equations in our model and their respective literature references can be found in S1 Table. For gene expression reactions, the Vmax value was calculated using one of two methods: (1) we converted specific activity to Vmax (nM/sec) by multiplying the specific activity of the enzyme, which was provided by the BRENDA Enzyme Database [42], by a factor that correlates to the molecular mass of the enzyme and the dynamic concentration of the species; or (2) using the slope of experimentally published enzyme activity graphs to calculate Vmax. Rate constants used in this model are in nanomolar units to stay consistent with previous models [11, 16]. However, in order to compare model outcomes to *in vitro* and *in silico* system dynamics, we report concentrations of the different species in this model in terms of relative expression to the control, which we define as the LPS-only stimulated model. Lastly, to capture the complexity of this system, this model includes intermediate signaling molecules, forward, reverse, and degradation reaction rates, and negative feedback inhibitory proteins that modulate the production of iNOS at various levels.

To modularize the modeling of the network, the signaling pathway was divided into four sections: (1) IFN-γ activated JAK/STAT signal transduction leading to STAT1 dimer formation, (2) LPS activated MAPK signal transduction and subsequent NF-κB activation and nuclear translocation, (3) AP1, IRF-1, TNF-α, and iNOS gene expression, and (4) metabolic production of nitric oxide and arginine, which is modulated by the NOS enzyme.

### IFN-γ Activation of JAK/STAT Pathway

We used the previously published JAK/STAT model by Yamada, et al. as a prototype for modeling the IFN-γ signaling pathway [16]. The modeling parameters for the JAK/STAT pathway are based on the curated version of the model reported in the Biomodels database [41]. After implementing the JAK/STAT model and verifying that our results were consistent with the Yamada model, we enhanced the original model by updating the volumetric representation of the nuclear and cytoplasmic compartments, which were both set to 1 um^3^ in the Yamada model. Using the volume of an alveolar macrophage, calculated as 4990 um^3^, we approximated the volume of the cytoplasmic volume as 2495 um^3^ and the nuclear volume as 499 um^3^[43]. These parameters were based on values from the BioNumbers database, and were derived based on the estimate that the cytoplasm of a cell accounts for approximately half of a cell’s volume and the nucleus accounts for ten percent of the cellular volume.

The JAK/STAT pathway has two translocation reactions, namely, the nuclear translocation of STAT1 phosphorylated dimers and cytoplasmic translocation of SOCS1 mRNA. We accounted for volumetric changes of both, STAT1 phosphorylated dimers from the cytoplasm to the nucleus and the nuclear translocation of SOCS1 mRNA from the nucleus to the cytoplasm. In Eq 1 the first term describes the nuclear translocation of the cytoplasmic STAT1c phosphorylated dimers, which is multiplied by a volumetric conversion. The second term is the formation of the dimer in the nucleus from its separate components and is therefore, a squared term. Eq 1 also includes the breakdown of the dimer in the nucleus. The last term describes the binding of the nuclear STAT1 phosphorylated dimers with a nuclear phosphatase PPN and its subsequent dissociation reaction.

$$\begin{array}{ll} \frac{d}{dt}STAT1nP\_STAT1nP & =\left(\frac{Vol_{cyt}}{Vol_{nuc}}\;\times \;k_{26}\times \;STAT1cP\_STAT1cP\left(t\right)\right)\\ & +\left(\left(k_{27}\;\times \;STAT1nP^{2}\left(t\right)\right)-\left(k_{28}\times \;STAT1nP\_STAT1nP\left(t\right)\right)\right)\\ & -\left(\left(k_{32}\times \;PPN\left(t\right)\;\times \;STAT1nP\_STAT1nP\left(t\right)\right)-\left(k_{33}\;\;\times \;\;PPN\_STAT1nP\_STAT1nP\left(t\right)\right)\right) \end{array}$$ (1)

In Eq 2 the first term describes the cytoplasmic translocation of the synthesized nuclear SOCS1 mRNA. This step is also multiplied by a volumetric conversion to account for translocation from the nucleus to the cytoplasm. The second term accounts for cytoplasmic mRNA degradation.

$$\frac{d}{dt}SOCS1\_mRNAc=\left(\frac{Vol_{nuc}}{Vol_{cyt}}\;\times \;SOCS1\_mRNAn\left(t\right)\right)-\left(k_{42}\times \;SOCS1\_mRNAc\left(t\right)\right)$$ (2)

### LPS-MAPK Pathway

**Fig 2:**
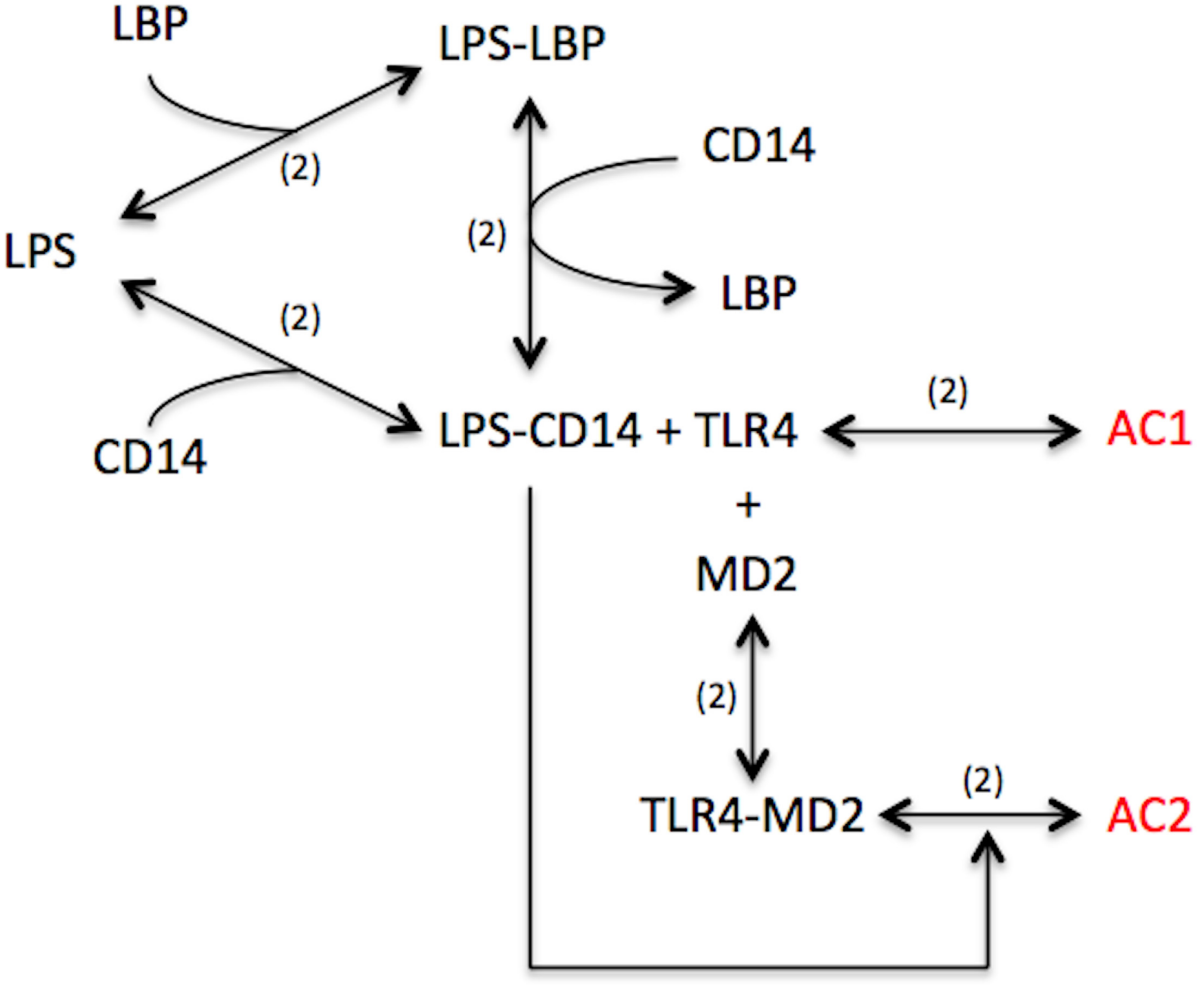
Reaction Scheme of LPS Mediated Membrane Complex Activation. Six reversible reactions capture the mechanism of LPS binding and activation of macrophages. The numbers in parenthesis denote the number of individual reactions implemented in the model to represent a specific binding/unbinding reaction. In short, LPS can activate macrophages in two ways, either TLR4 mediated or TLR4 and MD2 mediated, the latter of which is significantly faster and more robust.

The LPS-MAPK component of the model was derived from a 2007 paper by Shin et al on kinetics of LPS-TLR4 binding [44]. Studies on the kinetics of LPS activation via the MAPK pathway in macrophages indicate that LPS binds to CD14 through the aid of LPS binding protein (LBP) [45–47]. Although LPS can spontaneously diffuse from the bacterial membrane and bind with CD14, LBP can significantly enhance the association of the two. Once bound, LBP releases from the ternary complex and the resulting binary complex, LPS-CD14 activates the membrane bound Toll-like receptor 4 (TLR4) and MD2 complex (Fig 2) [47, 48]. Similar to the kinetics of LBP, LPS-CD14 bound to TLR4 can activate the MAPK pathway, however, the co-expression of MD2 significantly enhances the activation of the MAPK pathway [49]. LPS kinetics was modeled using ten derived ordinary differential equations capturing the mechanism outlined above.

- LPS
  $$\begin{array}{ll} \frac{d}{dt}LPS & =-\left(k_{77_{1}}\;\times \;LBP\left(t\right)\;\times \;LPS\left(t\right)\right)+\left(k_{77_{-1}}\;\times \;LBP\_LPS\left(t\right)\right)\\ & -\left(k_{77_{2}}\times \;LPS\left(t\right)\times CD14\left(t\right)\right)+\left(k_{77_{-2}}\;\times \;LPS\_CD14\left(t\right)\right)\\ & +\left(k_{77_{-3}}\times \;LPS\_CD14\left(t\right)\right) \end{array}$$ (3)
  The first term in Eq 3 represents the binding of LBP to LPS and thus LPS is depleted. The binary complex formed also has a reverse rate in which free LBP and LPS is replenished back into the system. Furthermore, LPS has a tendency to bind to CD14 without LBP but at a much slower rate. The formation of the LPS-CD14 binary complex is also reversible and thus, LPS can be replenished back into the system at the indicated reverse rate. Lastly, the binding of LBP-LPS to CD14 also results in the formation of the LPS-CD14 binary complex, which is also a reversible reaction leading to free LPS since LBP transfers LPS to CD14.
- LBP
  $$\begin{array}{ll} \frac{d}{dt}LBP & =-\left(k_{77_{1}}\;\times \;LBP\left(t\right)\;\times \;LPS\left(t\right)\right)+\left(k_{77_{-1}}\times \;LBP\_LPS\left(t\right)\right)\\ & +\left(k_{77_{3}}\;\times \;LPS\_LBP\left(t\right)\;\times \;\;CD14\left(t\right)\right) \end{array}$$ (4)
  Eq 4 shows similar binding kinetics as Eq 5. However, LBP is also replenished back into the system by the transferring of LPS to CD14. The initial concentration of LBP was set to equal the concentration of LPS, as LBP was shown to bind stoichiometrically one to one to LPS (47).
- LPS-LBP
  $$\begin{array}{ll} \frac{d}{dt}LPS\_LBP & =\left(k_{77_{1}}\;\times \;LBP\left(t\right)\;\times \;LPS\left(t\right)\right)\;-\;\left(k_{77_{-1}}\times \;LBP\_LPS\left(t\right)\right)\\ & -\left(k_{77_{3}}\;\times \;LPS\_LBP\left(t\right)\;\times \;\;CD14\left(t\right)\right) \end{array}$$ (5)
  Eq 5 represents complementary but inverse reaction kinetics to that of LPS and LBP. The binary complex formation for LPS-LBP is captured in the first term, with its reversible degradation in the second and its usage captured in the third term.
- CD14
  $$\begin{array}{ll} \frac{d}{dt}CD14 & =-\left(k_{77_{2}}\;\times \;\;LPS\left(t\right)\;\times \;CD14\left(t\right)\right)+\left(k_{77_{-2}}\;\times \;\;LPS\_CD14\left(t\right)\right)\\ & -\left(k_{77_{3}}\;\times \;\;LPS\_LBP\left(t\right)\;\times \;CD14\left(t\right)\right)+\left(k_{77_{-3}}\;\times \;LPS\_CD14\left(t\right)\right) \end{array}$$ (6)
  The first term in Eq 6 represents the association of LPS to CD14 and the generation of unbound CD14 from the breakdown of the LPS-CD14 complex. However, Shin et al pointed out that the association of LPS to CD14 without LBP is 10,000 slower than with [50]. Additionally, the equation captures CD14’s role as an acceptor of LPS transferred from LBP.
- LPS-CD14
  $$\begin{array}{ll} \frac{d}{dt}LPS\_CD14 & =\left(k_{77_{2}}\;\times \;\;LPS\left(t\right)\;\times \;\;CD14\left(t\right)\right)-\left(k_{77_{-2}}\;\times \;\;LPS\_CD14\left(t\right)\right)\\ & +\left(k_{77_{3}}\;\times \;LPS\_LBP\left(t\right)\;\times \;CD14\left(t\right)\right)-\left(k_{77_{-3}}\;\times \;LPS\_CD14\left(t\right)\right)\\ & -\left(k_{77_{5}}\;\times \;LPS\_CD14\left(t\right)\;\times \;TLR4\left(t\right)\right)+\left(k_{77_{-5}}\;\;\times \;AC_{1}\left(t\right)\right)\\ & -\left(k_{77_{6}}\;\;\times \;LPS\_CD14\left(t\right)\;\times \;TLR4\_MD2\left(t\right)\right)+\left(k_{77_{-6}}\;\;\times \;AC_{2}\left(t\right)\right) \end{array}$$ (7)
  The first two terms capture the reversible binding and unbinding of LPS to CD14 generating the binary complex. The third term represents the transfer of LPS from LBP-LPS resulting in free LBP. The fourth term however is not a reversible term; rather, it signifies an alternate unbinding reaction for LPS-CD14 governed by a different rate than the reversible binding rate of the second term. Lastly, the remaining four terms show the binding and unbinding of LPS-CD14 to membrane receptors, which generates either activated complex 1 or the more robust activated complex 2.
- TLR4
  $$\begin{array}{ll} \frac{d}{dt}TLR4 & =-\left(k_{77_{4}}\;\;\times \;TLR4\left(t\right)\;\times \;MD2\left(t\right)\right)+\left(k_{77_{-4}}\;\times \;TLR4\_MD2\left(t\right)\right)\\ & \;-\left(k_{77_{5}}\;\times \;LPS\_CD14\left(t\right)\;\times \;\;TLR4\left(t\right)\right)+\;\left(k_{77_{-5}}\;\times \;AC_{1}\left(t\right)\right) \end{array}$$ (8)
- MD2
  $$\frac{d}{dt}MD2=-\left(k_{77_{4}}\;\times \;TLR4\left(t\right)\;\times \;MD2\left(t\right)\right)+\left(k_{77_{-4}}\;\times \;TLR4\_MD2\left(t\right)\right)$$ (9)
- TLR4-MD2
  $$\begin{array}{ll} \frac{d}{dt}TLR4\_MD2 & =\left(k_{77_{4}}\;\;\times \;TLR4\left(t\right)\;\times \;MD2\left(t\right)\right)+\left(k_{77_{-4}}\;\times \;TLR4\_MD2\left(t\right)\right)\\ & -\left(k_{77_{6}}\;\times \;LPS\_CD14\left(t\right)\;\times \;\;TLR4\_MD2\left(t\right)\right)+\left(k_{77_{-6}}\;\times \;AC_{2}\left(t\right)\right) \end{array}$$ (10)
  Eqs 8–10 represents similar reaction mechanisms, but species are results of slightly varied binding and interaction cascades. Eq 8 quantitatively describes the usage terms for TLR4, including TLR4 binding to MD2 and to LPS-CD14. Eq 9 shows a similar usage term for MD2 binding to TLR4. The dynamics of the binary complex, TLR4-MD2, is represented in Eq 10, which shows the association rate then the activation rate by LPS-CD14 to generate activated complex 2.
- Activated complex 1
  $$\begin{array}{ll} \frac{d}{dt}AC_{1} & =\left(k_{77_{5}}\;\times \;LPS\_CD14\left(t\right)\;\times \;TLR4\left(t\right)\right)-\left(k_{77_{-5}}\times \;AC_{1}\left(t\right)\right)\\ & -\;\left(k_{79}\;\times \;AC_{1}\left(t\right)\;\times \;PI3K\left(t\right)\right) \end{array}$$ (11)
  The binding of LPS-CD14 to TLR4 generates activated complex 1 (AC1). This is captured as a reversible reaction. Activated complex 1 can initiate the MAPK pathway by irreversibly binding to PI3K [50, 51].
- Activated complex 2
  $$\begin{array}{ll} \frac{d}{dt}AC_{2} & =\left(k_{77_{6}}\;\times \;LPS-CD14\left(t\right)\;\times \;TLR4-MD2\left(t\right)\right)-\left(k_{77_{-6}}\times \;AC_{2}\left(t\right)\right)\\ & -\;\left(k_{79}\;\times \;AC_{2}\left(t\right)\;\times \;PI3K\left(t\right)\right) \end{array}$$ (12)
  The association of TLR4 to MD2 followed by the subsequent association with LPS-CD14 is needed for the formation of Activated Complex 2 (AC2). This quaternary structure can activate PI3K at an optimal rate, which is shown by the third term in Eq 12.

The activated complex (AC1 or AC2) proceeds to activate the MAPK pathway through an enzyme dependent, RAS independent manner. Activation proceeds through a phosphate transfer reaction that leads to the activation of the MAPK kinase system. These kinases are known by various names throughout literature and a list of synonymous names can be found in the BRENDA enzyme database [42]. To specifically model the JNK sub-pathway, we used a JNK specific nomenclature where TAK1 represents MAPKKK, SEK1 represents MAPKK, and JNK represents MAPK [10, 52]. Although the signal transduction leading from the activated complex to the activation and phosphorylation of JNK is a series of enzymes, this component of the signaling pathway was also modeled using mass action kinetics. Since the substrate concentration, or the preceding enzyme concentration does not exceed the subsequent enzyme concentration the Michaelis-Menten formulation for the kinetics was not used, as the assumption of substrate exceeding enzyme did not hold. Furthermore, the pathway represents a phosphate activation mechanism in which ATP provides the phosphate for each successive kinase to activate the next. As both phosphorylation and de-phosphorylation reactions were included within the model, ATP was assumed to be constant throughout the system and was not explicitly represented in the model. Equations that show the dynamics of the MAPK signal transduction can be found in S1 Table.

**Fig 3:**
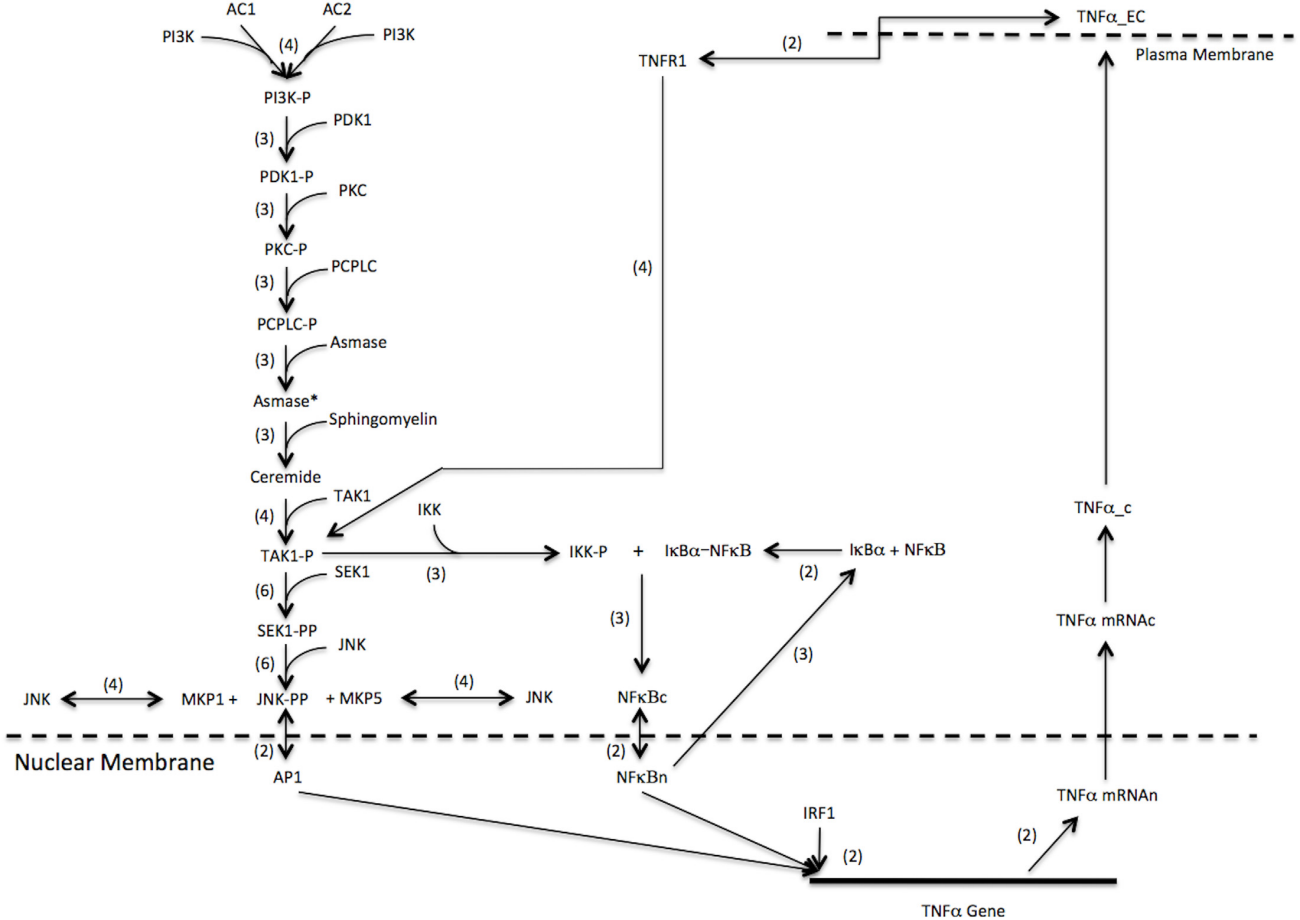
LPS Activated Cytokine Synergy on iNOS gene Expression and NO Production. LPS stimulation activates transcription factors AP1 and NF-κB, which in turn activates the TNF-α gene. Translated TNF-α exports to the extracellular compartment and activates the TNF-α-receptor 1 that proceeds to further stimulate AP1 and NF-κB via an autocrine loop. NF-κB can also activate IRF1, which increases production of TNF-α, however, the production of IRF1 is greatly increased by IFN-γ stimulation and STAT1 phosphorylated dimer activation (bold arrows). Therefore, the synergistic expression of TNF-α and IRF1 by IFN-γ greatly amplifies iNOS gene expression and NO production.

The downstream product of many proinflammatory-signaling pathways leads to the activation of NF-κB. This prominent transcription factor is locked in an inactive form in the cytosol by its inhibitor, IκB-α. The phosphoryl-activation of TAK1 leads to the activation of the enzyme IκB-Kinase (IKK) that causes the dissociation of IκB-α from NF-κB [14, 15]. To capture the regulatory mechanisms of the IκB-NF-κB complex formation and dissociation, we incorporated reaction rates and parameters from a model by Sharp et al. that described the effect of LPS stimulation on NF-κB activation. The Sharp model showed how inactive cytosolic NF-κB bound to IκB-α could dissociate and translocates into the nucleus where it acts as a transcription factor for numerous proinflammatory and cell regulatory genes including iNOS [11]. However, NF-κB concurrently activates the IκB-α gene and thus, provides a negative feedback mechanism to down regulate its own activation [53, 54]. This feedback mechanism represses the amount of free NF-κB during prolonged inflammatory responses. Our expanded model includes the binding of IKK to the inactive IκB-a-NF-κB complex, production rate of activated free NF-κB, nuclear translocation, gene expression of IκB-α, and nuclear regulatory reactions that eventually export free nuclear NF-κB back into the cytoplasm (Fig 3).

To regulate the strength of the proinflammatory response, there exists a series of MAPK phosphatases (MKPs) that regulate the signaling pathways. Their regulatory mechanisms are important for cell survival as prolonged inflammatory responses can lead to cell cytotoxicity. It was shown that under LPS stimulation, MKP1 and MKP5 are the prominent MAPK phosphatases that regulate the MAPK component of the signaling pathway. Both phosphatases dephosphorylate JNK-PP back into its inactive form [55–59]. Reaction rates for JNK-PP de-phosphorylation were modeled similar to the cytoplasmic phosphatases in the Yamada model.

### Autocrine-based TNF-α Production

TNF-α gene expression is activated by both LPS and IFN-γ, and regulated by NF-κB, AP1, and more recently determined, IRF1 [60]. Analysis of the TNF-α promoter region showed three binding sites for NF-κB, one for AP1, and two for IRF1. However, all NF-κB and AP1 binding sites were required to initiate transcription from LPS whereas only IRF1 binding sites were required to initiate transcription by IFN-γ [60–62]. Optimal expression was observed when all six binding sites were occupied [63]. The mechanism of TNF-α gene expression was modeled in two parts, namely, activation by AP1 and NF-κB, and activation by IRF1. Since the order of transcription factor binding was not specified in literature, our model assumed that there was an equal probability of binding amongst the transcription factors. We therefore modeled an NF-κB trimer binding first followed by AP1 binding for the activation by LPS. Eq 13 captures the dynamics of TNF-α gene expression.

$$\begin{array}{ll} \frac{d}{dt}TNFalpha\_mRNA & =\left(\frac{k_{141}\;\times \;\;NFkBn^{3}\left(t\right)\;\times \;AP1\left(t\right)}{k_{142}\times \;k_{143}+\;k_{143}\times \;NFkBn^{3}\left(t\right)\;\;+\;\;NFkBn^{3}\left(t\right)\;x\;AP1\left(t\right)}\right)\\ & -\left(\frac{k_{168}\times \;IRF1^{2}\left(t\right)}{\left(k_{169}\;\times \;\frac{1+IRF2n\left(t\right)}{KIirf2}\right)+\;IRF1n^{2}\left(t\right)}\right)-\left(k_{144}\times \;TNFalpha\_mRNA\left(t\right)\right) \end{array}$$ (13)

The first two terms in Eq 13 are modeled using rapid equilibrium kinetics. We treated the first term as a bi-substrate enzymatic reaction with NF-κB trimer represented as a single ternary substrate. Similarly, the second term shows activation by two IRF1 substrates; however, we modeled this activation as a uni-substrate enzymatic reaction by treating the two IRF1 substrates as a binary complex. We also incorporate inhibitory effects of IRF2 binding to IRF1 into the second term.

To capture the autocrine mechanism of TNF-α, our model accounted for the translation of TNF-α mRNA to protein and the subsequent extracellular export. Volumetric changes from the intracellular to the extracellular compartments were also considered in the extracellular transport (Eq 14). The extracellular volume used in this model was calculated to be 8000 um^3^. Since TNF-α can also behave as a paracrine signaling mechanism, the extracellular volume was estimated to be the volume occupied by two macrophages with an average dimension of 10 x 10 x 10 um [43]. Given that the cytoplasmic volume is about four times smaller than the extracellular volume, the concentration of TNF-α decreases as it is extracellularly transported. The decrease in extracellular concentration correlates to the physiological extracellular concentrations of TNF-α being in the picomolar and femtomolar range [64].

Since there is such low concentration of extracellular TNF-α, a high affinity TNF-α receptor was assumed. Grell et al determined that, of the two TNF-α receptors, TNF-α receptor 1 (TR1) is primarily activated during a proinflammatory response [64]. Following ligand binding to receptor, the silencer of the death domain of TNFR1 dissociates from the intracellular death domain of TR1. The dissociation of the silencer enables TNF receptor-associated death domain (TRADD) to bind the exposed death domain. TRADD subsequently recruits receptor-interacting protein (RIP), cellular inhibitor of apoptosis 1/2 (c-IAP), and ubiquitin conjugated protein (Ubc13), which together form the activated TNF-α -receptor complex [65]. However, experiments with TRADD knockout mice showed virtually no TNF-α activation, therefore, our model assumes that TRADD binding is essential to form the activated complex. Similar to LPS membrane activation, we used mass action kinetics to model the mechanisms involved in TNF-α activation (Eqs 14 to 18).

$$\frac{d}{dt}TNFalphaEC=\left(\frac{Vol_{cyt}}{Vol_{ext}}\right)\times \;\left(k_{146}\times \;TNFalphac\left(t\right)\right)$$ (14)

$$\frac{d}{dt}TR1=\;-\left(k_{147}\times \;TNFalphaEC\left(t\right)\;\times \;TR1\left(t\right)\right)+\left(k_{148}\times \;TNFR1\left(t\right)\right)$$ (15)

$$\begin{array}{ll} \frac{d}{dt}TNFR1 & =\left(k_{147}\;\times \;TNFalphaEC\left(t\right)\times \;TR1\left(t\right)\right)-\left(k_{148}\;\times \;TNFR1\left(t\right)\right)\\ & -\left(k_{151}\;\times \;TNFR1\left(t\right)\;\times \;TRADD\left(t\right)\right)+\left(k_{152}\;\times \;TNFR1\_TRADD\left(t\right)\right) \end{array}$$ (16)

$$\frac{d}{dt}TRADD=-\left(k_{151}\;\times \;TNFR1\left(t\right)\times \;TRADD\left(t\right)\right)+\left(k_{152}\;\times \;TNFR1\_TRADD\left(t\right)\right)$$ (17)

$$\begin{array}{ll} \frac{d}{dt}TNFR1_{TRADD} & =\left(k_{151}\;\times \;TNFR1\left(t\right)\times \;TRADD\left(t\right)\right)-\left(k_{152}\;\times \;TNFR1\_TRADD\left(t\right)\right)\\ & -\left(k_{153}\;\times \;TNFR1\_TRADD\left(t\right)\;\times \;TAK1\left(t\right)\right)\;–\left(k_{153b}\;\times \;TNFR1\_TRADD\left(t\right)\right) \end{array}$$ (18)

Eq 14 models the extracellular transport of TNF-α multiplied by a volumetric conversion. Extracellular TNF-α can then bind to the TR1 reversibly as shown in Eq 15. The activated complex forms with the binding of TNFR1 to TRADD generating a ternary complex (Eqs 16 to 18), which can then activate TAK1 (MAPKKK) leading to the activation of downstream MAPK pathway products.

### Expression of iNOS Gene Transcription Regulatory Factors

The iNOS gene is regulated by four transcription factors: AP1, NF-κB, STAT1 phosphorylated dimers, and IRF1. Of the four transcriptions factors, equations regulating gene expression were only derived for IRF1. AP1 gene expression was based on the empirical observation that AP1 levels were found to be proportional to the levels of nuclear JNK-PP [66, 67]. Therefore, we included a JNK-PP dependent AP1 production term and a nuclear translocation step for JNK-PP that accounts for volumetric changes. A kinetic model for NF-κB activation was previously published in the Sharp et al model and production terms for IκB-α were also included to regulate the levels of NF-κB [11]. We used the Sharp model for NF-κB and STAT1 phosphorylated dimers, as previously described, were modeled similar to the Yamada model.

Previously, IFN-γ was shown to be the only inducer of IRF1 gene expression, suggesting that nuclear STAT1 phosphorylated dimers contribute as transcriptions factors to the induction of the IRF1 gene [68–71]. However, further analysis of the IRF1 promoter region revealed one binding site for STAT1 phosphorylated dimers and another binding site for NF-κB. Furthermore NF-κB was found to activate IRF1 gene expression, however both transcription factors were required to initiate transcription at an optimal rate [17, 69–71]. To capture the full dynamics of IRF1 gene expression, the following equation was derived:

$$\frac{d}{dt}IRF1\_mRNA=\left(\frac{\left(k_{135}\;\times \;k_{137}\times \;NFkBn\left(t\right)\right)+\left(k_{135b}\times \;STAT1nP\_STAT1nP\left(t\right)\;\times \;NFkBn\left(t\right)\right)}{\left(\left(k_{136}\;\times \;k_{137}\right)\;\times \;\left(1+\;\frac{IRF2n\left(t\right)}{KIirf2}\right)\right)+\left(k_{137}\times \;NFkBn\left(t\right)\right)+\left(k_{136}\times \;STAT1nP\_STAT1nP\left(t\right)\right)+\left(STAT1nP\_STAT1np\left(t\right)\;\times \;NFkBn\left(t\right)\right)}\right)$$ (19)

The numerator of Eq 19 shows that NF-κB alone can activate transcription however, optimal expression of the IRF1 gene occurs when both transcription factors are present.

### iNOS Gene Expression

Although four transcription factors regulate iNOS gene transcription, six binding sites exist within the promoter region of the gene. Two belong to AP1 (distal and proximal), two to NF-κB (distal and proximal), one to IRF-1, and one to the STAT1 dimer [52]. The order of binding mirrors the synergistic effect of the IFN-γ and TNF-α pathways. Order-dependent variation was evidenced in knock out experiments of distal and proximal regions, which resulted in differences in gene expression [72]. These knockout experiments suggested that binding of transcription factors to the distal AP1 binding region (nucleotide-1069) and the proximal NF-κB region (nucleotide-86) along with IRF-1 enables the formation of the initial looping of the iNOS promoter region, creating a favorable confirmation for RNA polymerase to bind. Although IRF1 was needed for initial transcription, STAT1 dimers were not as knockout experiments did not completely diminish iNOS mRNA expression rather reduced the expression levels of iNOS. Therefore, STAT1 dimers and TNF-α stimulation both contributed an additive effect to gene expression but were not essential to the mechanism. These observations were used to derive a more representative rate equation for quantitative modeling of iNOS gene expression [63, 73, 74].

**Fig 4:**
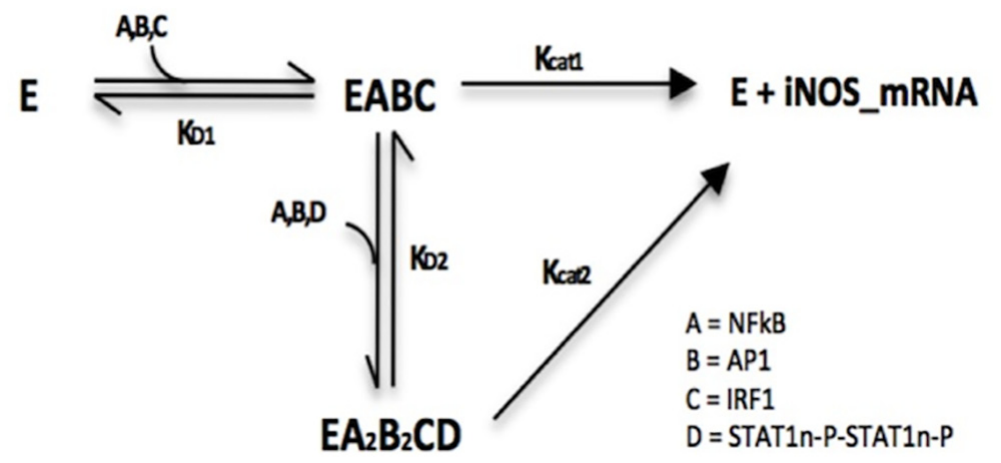
iNOS Gene Expression Mechanism. The Enzyme term in this figure refers to the DNA-RNA Polymerase complex. Dissociation constants are labeled accordingly and their values can be found in the S1 Table.

To capture the initial transcriptional impact of IRF1, NF-κB, and AP1 and the compounding effect of STAT1 dimers, we assumed in our model that the binding of IRF1, NF-κB, and AP1 to sites on the iNOS promoter occurred first. The formation of this primary intermediate complex resulted in a minimal rate of iNOS mRNA formation (Kcat1). The remaining three binding sites for AP1, NF-κB, and STAT1 dimers can then be occupied once the primary intermediate complex is formed. This fully occupied promoter complex resulted in an optimal gene expression rate (Kcat2). The iNOS reaction network can be seen in Fig 4. From the reaction scheme, the following rate equation for gene expression was derived:

$$\frac{d}{dt}iNOS\_mRNA=\frac{vmax_{1}\times \;K_{D1}\;\times \;NFkBn\left(t\right)\;\times \;AP1\left(t\right)\;\times \;IRF1\left(t\right)\;+\;\;vmax_{2}\times \;NFkBn^{2}\left(t\right)\;x\;AP1^{2}\left(t\right)\;\times \;IRF1\left(t\right)\;\times \;STAT1nP\_STAT1nP\left(t\right)}{K_{D1}K_{D2}\;\;+\;K_{D2}\;\times \;NFkBn\left(t\right)\;\times \;AP1\left(t\right)\;\times \;IRF1\left(t\right)\;\;+\;\;NFkBn^{2}\left(t\right)\;\times \;AP1^{2}\left(t\right)\;\times \;IRF1\left(t\right)\;\times \;STAT1nP\_STAT1nP\left(t\right)}$$ (20)

The numerator in Eq 20 contains two terms to represent both transcription factor complexes. The first term contains the three transcription factors that are needed to initiate transcription of the iNOS gene. The second term contains all the transcription factor variables needed for the fully occupied promoter binding site complex. Accordingly, the Vmax of the second term is higher than that of the first accounting for the difference between optimal and sub-optimal gene expression levels. The kinetic parameters in the denominator of Eq 20 were derived similar to Eqs 13 and 19 and represent the dissociation constants for the formation of the intermediary complexes.

### Proinflammatory Effector Response

The ultimate outcome of the activation of LPS, TNF-α, and IFN-γ associated signal transduction pathways in our model is the temporal expression of the iNOS enzyme and the concomitant production of nitric oxide (NO). NO is a critical immune response molecule that directly contributes to the production of bactericidal effectors against intracellular pathogens. Following the expression of iNOS mRNA, we modeled cytoplasmic translocation of nuclear iNOS mRNA and subsequent translation using mass action kinetics. We used Michaelis-Menten kinetics to model metabolic production of NO. The production of NO is regulated by the arginine-citrulline cycle as iNOS obtains a nitrogen group from L-arginine creating L-citrulline. In addition to iNOS, the cycle uses two enzymes, arginosuccinate synthase and arginosuccinate lyase to replenish the amount of arginine needed to drive the iNOS reaction [75, 76]. Our model assumed the amount of arginine sufficiently exceeds the concentration of iNOS and thus, the use of the Michaelis-Menten form was valid.

Eq 21 represents the iNOS catalyzed production of NO using arginine as a substrate.

$$V_{NO}=\;\frac{vmax\;\times \;arginine\left(t\right)}{K_{Marg}+arginine\left(t\right)}$$ (21)

The arginine-citrulline cycle modeled in Eqs 22 and 23 is regulated by two enzymes, arginosuccinate synthase and arginosuccinate lyase.

$$V_{arginosuccinate}=\;\frac{vmax\;\times \;citrulline\left(t\right)}{K_{Mcit}+citrulline\left(t\right)}$$ (22)

$$V_{arg}=\;\frac{vmax\;\times \;arginosuccinate\left(t\right)}{K_{Margsuc}+arginosuccinate\left(t\right)}$$ (23)

Eq 22 models the mechanism by which arginosuccinate synthase uses citrulline, a byproduct of NO formation by iNOS, to synthesize arginosuccinate. In Eq 23 arginosuccinate lyase then uses arginosuccinate to replenish arginine, which is used again as a substrate by iNOS. The combination of Eqs 21–23 represents the basic mechanism of the intracellular arginine-citrulline cycle. The Vmax and Km values for iNOS, arginosuccinate synthase, and arginosuccinate lyase were obtained from the BRENDA enzyme database [42]. The schematic of this cycle is also included in Fig 3 and the dynamics of this cycle are shown in S4 Fig.

### Sensitivity Analysis and Optimization

To determine the drivers of the model, we used the freely available software DAKOTA (Sandia National Laboratories) to perform sensitivity analysis (SA) and optimization on model parameters [77]. Although optimizing the entire system to a dataset of interest may result in a better correlation between theoretical and empirical results, it was more feasible to optimize over parameters that are statistically significant drivers of the model. We first performed sensitivity analysis to determine the statistically significant parameters with respect to iNOS production, and then performed optimization for the significant variables using a genetic algorithm.

We calculated partial regression correlation coefficients (PRCC) based on the outcome of 350 iterations of our model where each of the 183 model parameters were varied. The coefficients obtained from our simulation were compared against a statistically significant range determined by a PRCC that was calculated by the Student’s T-test to see which parameters were significant. We used a p-value of 0.001 to obtain a T-statistic in order to calculate the PRCC non-significance range, which equated to be ±0.253. The data from the SA was compiled into a 183 x 114 matrix where the rows represented the parameters and the columns represented the species in the model. If the parameters were statistically significant throughout the system, a value of 1 was placed at the respective row and column of the matrix. The scored rows were summed into a column matrix and if the value of each respective row was greater than 30, that is, appeared statistically significant more than 30 times throughout the model, it was deemed sensitive. Out of 183 parameters, 29 parameters appeared to be the main drivers of the model (Table 1).

**Table 1:** Significant Parameters that Drive the System.

Parameter	Description	Original Value	Optimized Value	Units	Sensitivity
k77_4_	TLR4 binding to MD2	7.50E-06	6.47E-06	1/nM*s	++
k77_-4_	Dissociation of TLR4-MD2	4.70E-04	3.65E-04	1/s	-
k77_5_	Association of LPS-CD14 to TLR4	3.23E-06	2.33E-06	1/nM*s	+
k77_-5_	Dissociation of LPS-CD14-TLR4	0.0454	6.34E-02	1/s	-
k77_6_	Association of LPS-CD14 to TLR4-MD2	3.23E-04	4.37E-04	1/nM*s	++
k77_-6_	Dissociation of LPS-CD14-TLR4-MD2 complex	0.0454	4.03E-02	1/s	-
k79	Association of AC1 to PI3K	3.85E-04	4.80E-04	1/nM*s	++
k81	Phosphorylation of PI3K to PI3K-P	3.85E-04	2.82E-04	1/s	+
k82	Association of PI3K-P to PDK1	6.43E-05	7.56E-05	1/nM*s	
k84	Phosphorylation of PDK1	6.43E-04	6.84E-04	1/s	+
k88	Association of PKC-P to PCPLC	1.83E-04	2.08E-04	1/nM*s	+
k96	Association of ceramide to TAK1	3.30E-04	3.30E-04	1/nM*s	
k97	Degradation of ceremide-TAK1 complex	1.80E-03	2.17E-03	1/s	—
k98	Phosphorylation of TAK1 to TAK1-P	0.005	7.40E-03	1/s	++
k99	Association of TAK-1 to SEK1	2.30E-04	2.57E-04	1/nM*s	+
k101	Phosphorylation of SEK1 to SEK1-P	0.005	5.64E-03	1/s	+
k102	Association of TAK1-P to SEK1-P	2.30E-03	2.70E-03	1/nM*s	+
k124	Association of TAK1-P to IKK	1.20E-04	8.93E-05	1/nM*s	—
k128	Degradation of IκB a-NF-κBc	5.00E-04	3.96E-04	1/s	—
k132	Random dissociation of IκB-a-NF-κBc complex	2.25E-05	2.61E-05	1/s	++
k135	Vmax IRF1 by NF-κB	0.003	4.43E-03	nM/s	++
k136	Dissociation constant Kirf1 of NF-κB binding to DNA	3.4	1.94E+00	nM	—
k138	IRF1 mRNA nuclear to cytoplasmic translocation	0.001	1.36E-03	1/s	++
k144	TNF-α mRNA nuclear to cytoplasmic translocation	0.001	8.05E-04	1/s	+
k164	Gene expression of IκB-a-mRNA	0.0165	1.47E-02	1/nM*s	—
k168	Vmax TNF-α by IRF1	2.00E-03	1.38E-03	nM/s	++
k169	Dissociation constant of IRF1 on TNF-α gene DNA	4.00E-03	4.99E-03	nM	-
k174	Nuclear translocation of IRF2c to IRF2n	0.005	4.60E-03	1/s	—
KIirf2	Inhibitory dissociation constant of IRF2 on IRF1	300	364.36	nM	++

Using the freely available software, DAKOTA, sensitivity analysis was performed on the model’s 183 parameters. 29 parameters were shown to be sensitive to 30 or more species within the model and therefore, they were considered to be significant drivers of the model and ultimately iNOS gene expression. Therefore, optimization was performed over these 29 parameters. Both original and optimized values of the parameters are included in the table along with their statistical significance and correlation to iNOS gene expression.

In addition to providing values for the drivers of our system, Table 1 also includes the level of significance each parameter has on the production of iNOS mRNA. Parameters whose PRCC was greater than 0.253 or less than -0.253 were deemed significant. To further distinguish the attributes of the drivers of the system, we assigned a plus sign to those parameters that show a positive correlation to the production of iNOS (0.253 ≤ *PRCC* < 0.6) and accordingly, a double plus sign that showed a statistically strong positive correlation (0.6 ≤ *PRCC* ≤ 1). We assigned a minus sign to those parameters that show a negative correlation to the production of iNOS (−0.253 ≥ *PRCC* > 0.6) and similarly, a double minus sign that showed a statistically strong negative correlation with the production of iNOS mRNA (−0.6 ≥ *PRCC* ≥ 1). Negative correlation was generally seen in parameters that were involved in dissociation and or degradation of dimerized species such as those included in the MAPK intermediates as seen with k97 and k128. Positive correlations were seen in parameters that were involved in association reactions that impact iNOS mRNA production.

For optimization, we used the genetic algorithm (GA) method included in DAKOTA, which is a heuristic process that evolves individual species (parameters) to produce a model output that fits the empirical solution with minimal error. We optimized our model to experimental values of iNOS mRNA obtained under LPS stimulation as reported by Mustafa et al [78]. We extrapolated and digitized the data from Mustafa et al. and normalized it in order to compare their empirical data and our simulation output in terms of relative expression. Although our initial simulations were similar to empirical results, our optimized model gave a better fit in terms of temporal dynamics of the system (S1 Fig). Optimized values of the 29 significant parameters and their corresponding functions are listed in Table 1.

## Results and Discussion

Using our model, we simulated the temporal production of iNOS mRNA, nitric oxide expression, and iNOS pathway intermediates in the presence of various stimulatory conditions to determine the effects of TNF-α and IFN-γ activation, and priming on host response to LPS stimulation. To stimulate or induce stimulation in terms of the model means to impose a non-zero predefined initial condition or value for the stimulatory species of interest (S2 Table lists all initial values used in the model). Out of a total of ten stimulatory conditions, seven were activating conditions, where the specific activating specie(s) was given a predefined initial value at simulation time t = 0, and three were priming conditions. To simulate priming, the cytokine specie(s) used to prime the system was given a predefined initial value and we simulated the 24-hour (simulation timescale) priming response of the system in the absence of LPS. We then followed the priming simulation with an 8-hour simulation in the presence of LPS, with the initial value of LPS set to a predefined value and all other species’ values remained at their post-priming levels. The seven activating conditions include activation by LPS (which was used as a control in the model), TNF-α, IFN-γ, TNF-α/IFN-γ, TNF-α and LPS, IFN-γ and LPS, and lastly, TNF-α/IFN-γ and LPS. The three priming conditions include priming by TNF-α, IFN-γ, and both, TNF-α /IFN-γ followed by LPS activation. We simulated host response for eight hours to remain consistent with previous models and with time periods used for *in vitro* experiments [78], which should allow our model to capture the expression dynamics of pathway intermediates and genes in our model.

### Activation of iNOS by LPS, TNF-α, and IFN-γ

**Fig 5:**
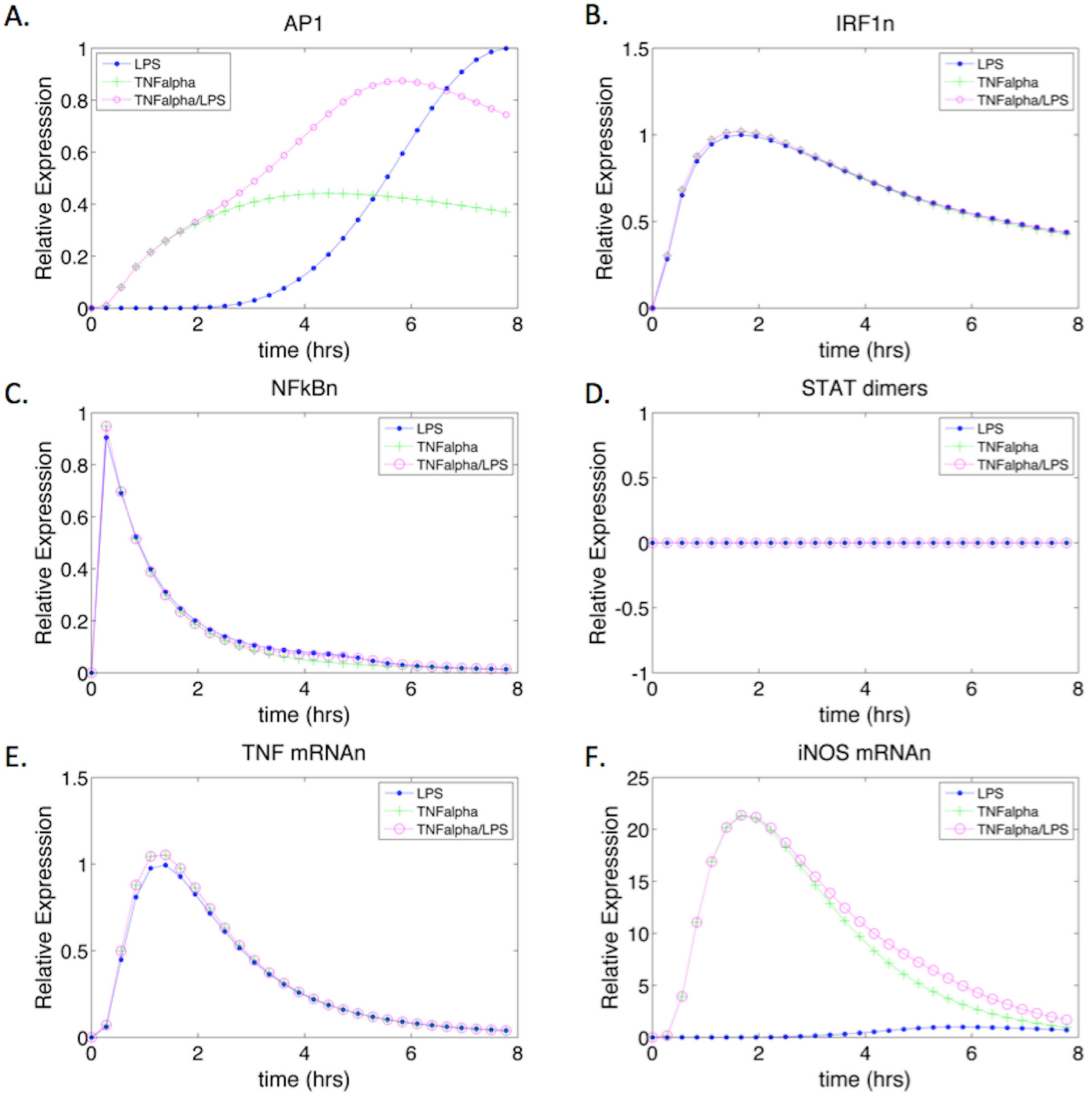
TNF-α Stimulated iNOS mRNA and Transcription Factor Expression Dynamics. The system was simulated with LPS (control), TNF-α, and TNF-α with LPS as inputs. (A-D) show the transcription factors of iNOS under the three stimulatory conditions. STAT1 phosphorylated dimers remain at zero since there is no IFN-γ stimulation. (E) Shows the expression of iNOS under LPS stimulation only. The delay in iNOS expression correlates to LPS causing a delayed activation of iNOS in the absence of other cytokines due to a larger activation cascade. (F) iNOS mRNA gene expression under TNF-α and TNF-α/LPS stimulation respectively.

We compared the dynamic impact of LPS and TNF-α activation on iNOS expression (Fig 5). The expression level of AP1 under LPS stimulation followed a similar trend as the iNOS mRNA expression for the control (Fig 5A and 5E). Since LPS stimulation acts further upstream in the MAPK cascade, requiring the activation of a longer signal transduction cascade, the depletion of AP1 occurred at a later time when compared to the AP1 dynamics seen under TNF-α stimulation. The faster activation of AP1 under TNF-α stimulation results from the cytokine’s ability to act on species further downstream in the signaling cascade. The TNF-α membrane complex can directly activate the MAP kinase pathway, bypassing the upper activation pathway. However, TNF-α stimulation resulted in a slightly reduced level of AP1 expression, which may be a result of the slightly increased expression of TNF-α mRNA [64]. Interestingly, the expression dynamics of AP1 under both, TNF-α and LPS co-stimulation represented a combination of the AP1 expression seen with TNF-α or LPS alone (Fig 5A).

Nuclear NF-κB reached peak expression levels relatively quickly after stimulation by LPS or TNF-α (Fig 5C). The rapid depletion of NF-κBn can be attributed to its usage in multiple gene expression reactions including AP1, IRF1, IκB-a, TNF-α, and iNOS. Furthermore, although the signal transduction reactions that lead to the activation of cytoplasmic NF-κB are relatively the same as those for the expression of AP1, the nuclear translocation rate of free cytoplasmic NF-κB is greater than that of phosphorylated JNK producing AP1 [15]. NF-κB expression dynamics appeared comparable under the three activating conditions, which may have resulted from a combination of interacting regulatory factors such as nuclear phosphatases and IκB-a gene expression. Under higher stimulatory conditions, regulatory factors that deplete free NF-κB were also expressed at higher levels and thus, help modulate the levels of NF-κB irrespective of LPS versus TNF-α activation as observed in our model (S2 Fig).

Although IRF1 initially was thought to be expressed by IFN-γ only, literature has suggested that NF-κB can also induce IRF1 gene expression [69]. Our model captures the production of IRF1 transcription factor during both, LPS and TNF-α stimulation. However, the levels of induction are similar under all three stimulatory conditions (Fig 5B). Unlike IRF1, STAT1 phosphorylated dimers are only produced due to the activation of the JAK/STAT membrane complex by IFN-γ. Thus, the levels of STAT1 phosphorylated dimers were zero during activation with LPS and/or TNF-α stimulation (Fig 5D). Consistent with our iNOS gene expression model (Fig 4), we observed that AP1, IRF1, and NF-κB were sufficient to induce iNOS transcription even in the absence of STAT1 dimers. LPS and/or TNF-α were therefore sufficient to induce iNOS (Fig 5E and 5F), with peak iNOS expression under TNF-α stimulation occurring nearly four hours earlier and producing twenty times more iNOS mRNA. The early expression levels of AP1 under TNF-α stimulation seem to drive this outcome since the levels of NF-κB and IRF1 are similar under all three conditions.

**Fig 6:**
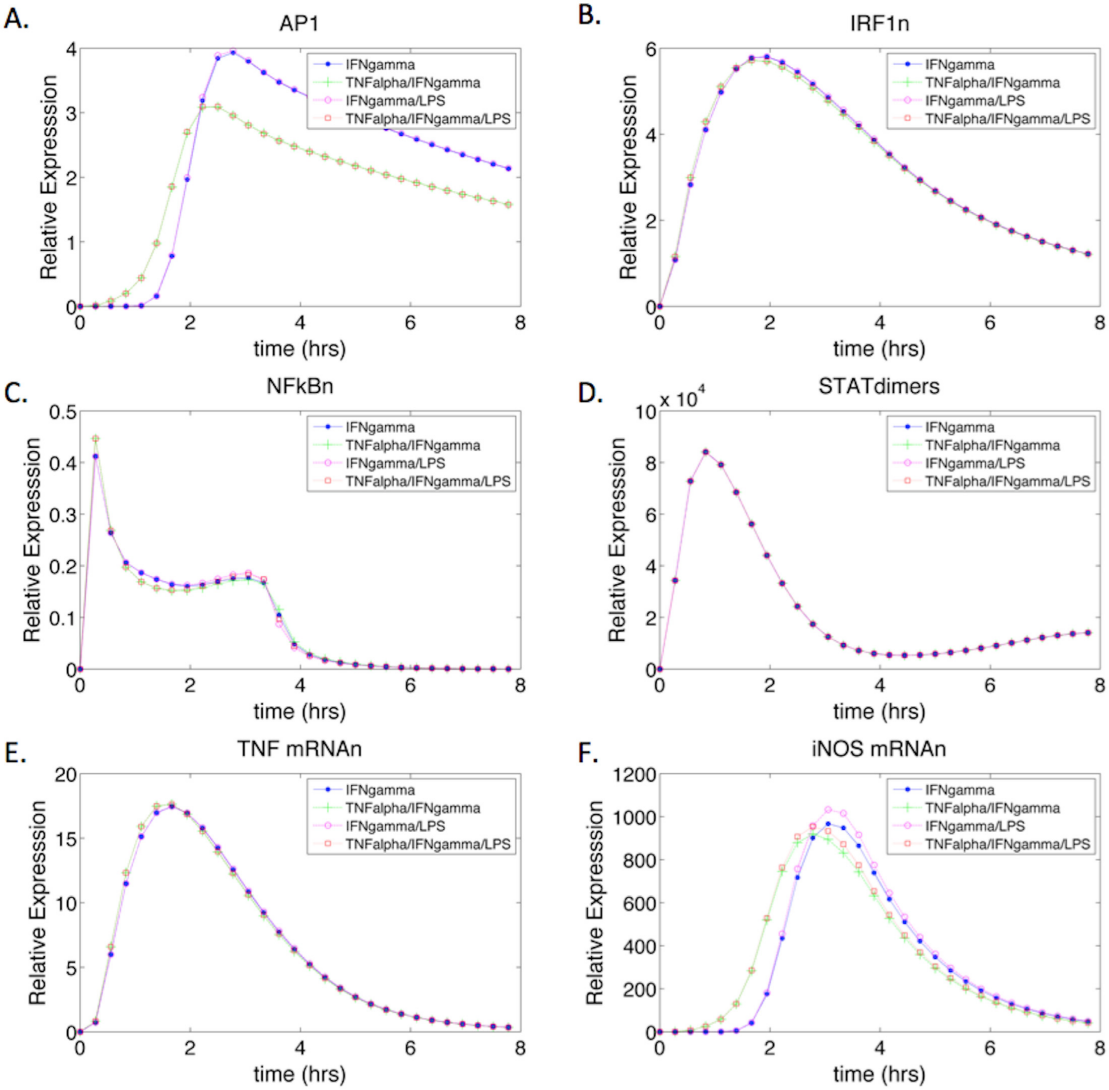
IFN-γ Stimulated iNOS mRNA and Transcription Factor Expression Dynamics. The system was stimulated with IFN-γ, IFN-γ /TNF-α, IFN-γ and LPS, and IFN-γ /TNF-α and LPS as inputs. (A-D) show the transcription factors of iNOS under the four stimulatory conditions. STAT1 phosphorylated dimers are expressed during IFN-γ stimulation (D) and the dynamics are similar to previous models [16]. (E) Shows the expression of TNF-α mRNA under IFN-γ stimulation. IFN-γ by itself is able to activate TNF-α gene expression through IRF1. (F) iNOS mRNA gene expression under the four stimulatory conditions. The magnitude of expression is much greater under IFN-γ stimulation as opposed to TNF-α only activation (Fig 4F), which may be attributed to the activation and compounding effect of TNF-α gene expression in the presence of IFN-γ.

The addition of IFN-γ caused the dynamics of the transcription factors and iNOS mRNA to follow slightly different trends than observed in TNF-α/LPS activation (Fig 6) for the remaining non-priming conditions: IFN-γ, TNF-α/IFN-γ, IFN-γ/LPS, and TNF-α /IFN-γ and LPS. Under all four activation conditions, AP1 followed similar expression dynamics however, when TNF-α was present the peak expression level of AP1 was slightly reduced but time to peak expression occurred earlier (Fig 6A). Contrastingly, in the absence of TNF-α (during IFN-γ and/or IFN-γ with LPS stimulation), there was a higher level of AP1 peak expression, but time to peak was slightly delayed. These dynamics highlight the role of TNF-α in the early induction of AP1 activity through direct activation of downstream components of the MAPK pathway, whereas activation of MAPK as a result of IFN-γ stimulation results in delayed AP1 activation. However the magnitude of activation is notably greater in the presence of IFN-γ (Figs 5A and 6A).

The expression of IRF1 and STAT1 dimers are more pronounced in the presence of IFN-γ (Fig 6B and 6D compared to Fig 5B and 5D) and their dynamics are comparable in all stimulatory conditions, suggesting that IFN-γ stimulation has a more prominent effect on these transcription factors than TNF-α alone. The ability of IFN-γ to induce a more robust proinflammatory response can be correlated to the increased levels of TNF-α mRNA expression (Fig 6E) and the compounding effects of the inflammatory TNF-α cytokine. Under IFN-γ stimulation, TNF-α mRNA levels are about 20 fold higher than levels observed under TNF-α stimulation. The increase in TNF-α gene expression can be correlated to the increased levels of IRF1, and the increase in NF-κB and AP1 produced by autocrine activation of the MAPK pathway by TNF-α. Ultimately, under IFN-γ stimulation, there is a collective increase in TNF-α, IRF1, AP1, and STAT1 phosphorylated dimers that all contribute to the increased magnitude in the proinflammatory response when compared to TNF-α stimulation.

### Characterizing the iNOS Expression Dynamics

**Fig 7:**
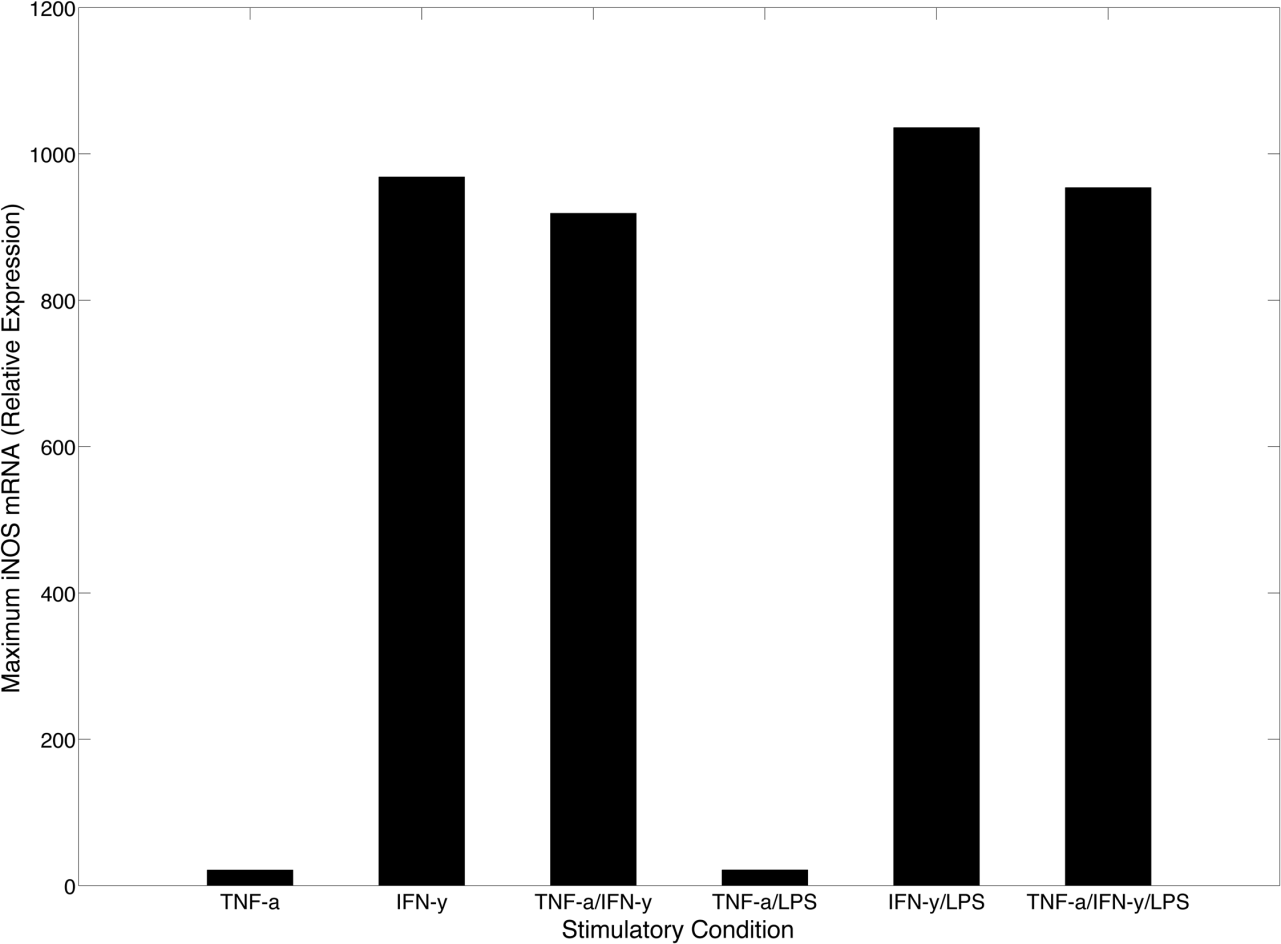
Maximum iNOS Expression. Maximum iNOS mRNAn values were obtained through model simulation under various conditions. Expression levels are relative to LPS control stimulation. The greatest increase in maximum iNOS production can been seen between TNF-α stimulation and IFN-γ stimulation. However, in the presence of LPS, there is only a slight increase in iNOS gene expression when compared to cytokine activation levels.

We used our model to comparatively characterize differences in maximum iNOS gene expression as a function of cytokine activation with or without LPS stimulation. Although TNF-α has the ability to activate the expression of iNOS during the initial infection, the need for IFN-γ mediated proinflammatory response is highlighted in Fig 7 where the activation levels of iNOS gene expression are significantly higher in the presence of IFN-γ suggesting that IFN-γ is a stronger cytokine-based activator of the proinflammatory response.

**Fig 8:**
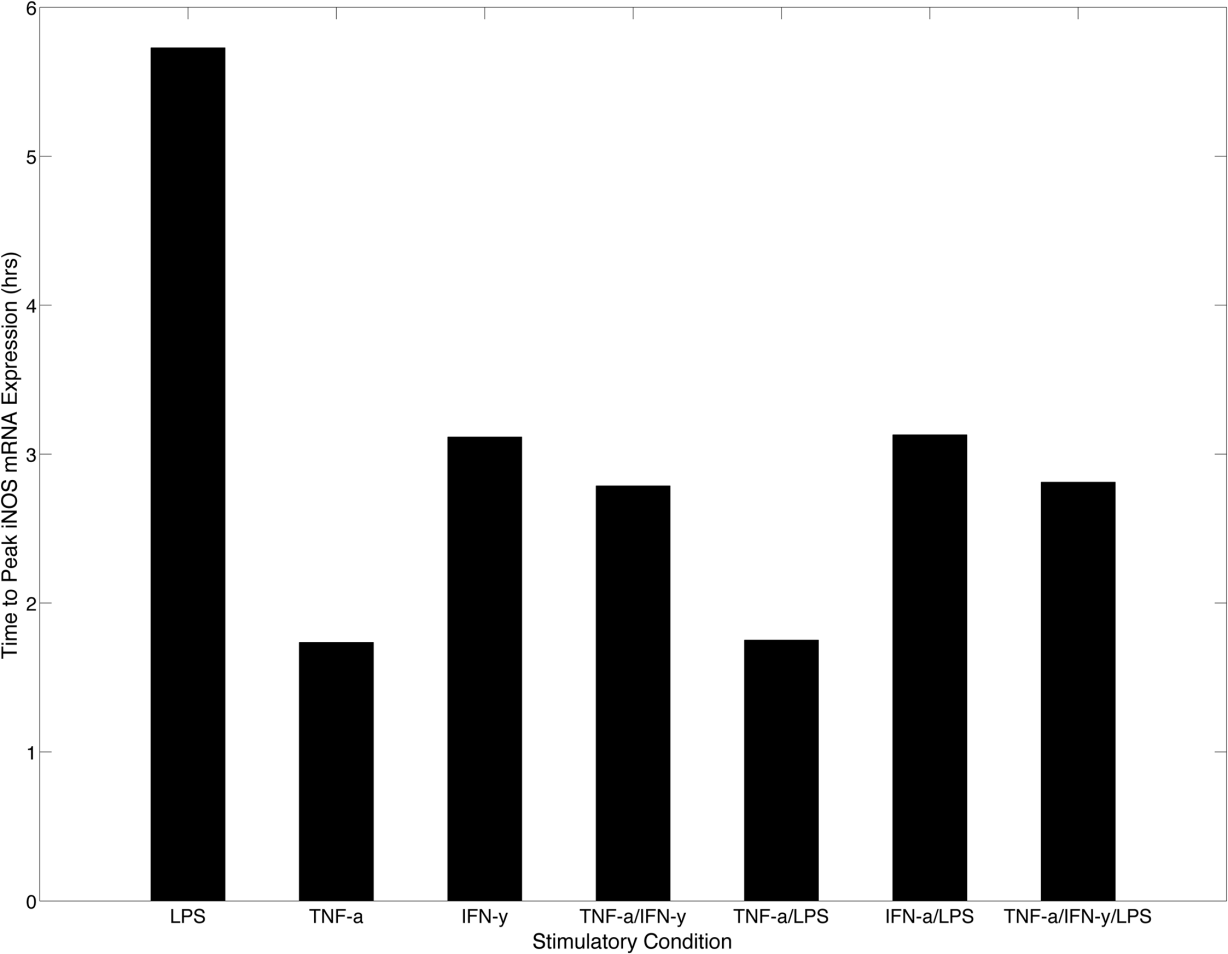
Time to Peak iNOS mRNA Expression. The model was simulated under the seven activation conditions and the time to peak iNOS gene expression was obtained for each simulation. The bar graph suggests that LPS is the slowest activator of iNOS whereas TNF-α is the fastest. This feature highlights the need for autocrine regulation of TNF-α during the proinflammatory response to help fight intracellular pathogens. IFN-γ however shows a delayed response when compared to TNF-α. This delayed response may be beneficial to boost the proinflammatory response following TNF-α stimulation.

Although the results of our model indicate that IFN-γ is a significantly more potent activator of the proinflammatory response (Fig 7), it has a longer delay to reach peak iNOS expression as evidenced in Fig 8 where the time to peak iNOS expression is shown for all stimulatory conditions. We observed that TNF-α was the fastest inducer of iNOS gene expression since both TNF-α and TNF-α with LPS stimulation required the least amount of time to reach peak iNOS expression (< 2 hours). This relatively short time-to-peak correlated strongly with results obtained by Lipniacki et al, which suggested that the amount of TNF-α Receptor 1 (TR1) far exceeds the amount of TNF-α found in the environment of activated or infected macrophages. The high receptor to ligand ratio thus enables a low concentration of TNF-α to have a high probability of binding to macrophages, generating a rapid response by circumventing the upstream signal transduction intermediates needed in LPS only stimulation [53]. Therefore, the rapid expression dynamics under TNF-α and LPS stimulation can be attributed to TNF-α and not the co-stimulation of LPS.

Under IFN-γ stimulation, the time to peak for iNOS mRNA expression is moderately reduced, which is likely due to the IFN-γ mediated TNF-α activation. Since IFN-γ activates TNF-α gene expression, the eventual autocrine mechanism for production of TNF-α likely contributes to the reduced time to initiate iNOS gene expression during IFN-γ stimulation. Interestingly, the indirect contribution by TNF-α reduces and brings the time to peak value under IFN-γ stimulation closer to the time to peak value seen under TNF-α only stimulation, which peaks over an hour earlier. This reduction highlights the need for TNF-α to be a first line, innate response cytokine since the time to peak under LPS stimulation is almost three times longer than TNF-α only stimulation and two times longer than IFN-γ only stimulation. However, in the co-stimulatory system where both IFN-γ and TNF-α were used to activate the system, iNOS gene expression achieved a slightly earlier time-to-peak with a large increase in iNOS expression at two hours post activation due to exogenously added TNF-α combining with TNF-α induced by IFN-γ to activate more rapid iNOS production (Fig 6F). These distinct dynamic mechanisms captured by our model, time and amplitude modulation of iNOS expression, can be correlated to the *in vivo* immune response cascade and the impact of reduced IFN-γ levels during the initial proinflammatory response. Chemokines secreted from the infected macrophage are required to recruit NK cells, a primary producer of IFN-γ during the innate immune response, to the site of infection. The time delay associated with NK cell migration to the site of infection and corresponding delay in significant levels of IFN-γ, necessitates a compensatory mechanism that can trigger a protective response. The fast response time of TNF-α may serve as a compensatory mechanism, given the contribution of the cytokine to the production of beneficial effector molecule used by macrophages to contain early infection. Upon arrival of NK cells, the availability of IFN-γ and the concomitant production of high levels of iNOS are required to clear infection [79–81].

### Investigating the Impact of Priming

Although macrophages and other immune cells such as NK cells secrete cytokines in response to cellular stress upon pathogenic infection, neighboring immune cells that are not infected have an increased likelihood of being primed by cytokines in their surrounding microenvironment. TNF-α secreted from the initially infected macrophage and IFN-γ secreted from recruited NK cells prime cells adjacent and to the periphery of the infected cell’s microenvironment respectively [82, 83]. Therefore, we used our model to investigate the dynamics of iNOS gene expression and NO production under cytokine priming. To simulate the effects of priming, we relied on experimental designs that were used to measure the effects of priming in macrophages infected with various pathogens. Variables derived from *in vitro* studies consisted of the concentration of the particular cytokine and the duration of priming time [34, 84–86]. To emulate the 24 hour priming condition, we set initial values for extracellular TNF-α concentration to 0.05 nM and initial IFN-γ concentration was set to 1 nM [82, 87]. After 24 simulation hours, the final concentrations of iNOS transcription factors, iNOS mRNAn, NO, and intracellular TNF-α, were used as starting concentrations for a subsequent 8-hour simulation with LPS stimulation. We assume that the primed values of these particular species are viable initial values under LPS stimulation and provide a good approximation of the priming effect. Furthermore, as our model does not contain any repletion terms for intermediary proteins that are involved in the signal transduction pathways, their levels are depleted during the priming period and are reset back to their initial values for subsequent LPS stimulation. Table 2 shows the peak expression levels of iNOS protein and NO production in a comparison between stimulatory and priming conditions.

**Table 2:** Peak and Average iNOS and NO Expression Levels.

Stimulatory Condition	Peak iNOS Protein Expression (Relative Expression)	Peak NO Expression (Relative Expression)
TNF-α + LPS	18	28
IFN-γ + LPS	804	940
TNFa/IFNy + LPS	789	974
TNFa primed LPS	2.7 x 10^5^	1.2 x 10^6^
IFNy primed LPS	7.1 x 10^7^	2.0 x 10^8^
TNFa/IFNy primed LPS	6.5 x 10^8^	1.9 x 10^9^

Peak iNOS protein and NO expression levels were obtained by taking the maximum value of the temporal expression levels of iNOS and NO under different stimulatory conditions using our model.

Results suggest that priming gives the uninfected macrophage a tremendous advantage against future pathogenic infections [88]. Under non-primed conditions, IFN-γ activation resulted in an order of magnitude greater NO production than TNF-α activation. Activation with both cytokines resulted in only a slight increase in NO expression when compared to IFN-γ activation despite the fact that there was a reduction in the peak iNOS protein levels. While the addition of TNF-α seems to slightly reduce the peak iNOS expression, earlier production of iNOS (Fig 8) may allow the system to achieve an overall higher concentration of NO than when activated by IFN-γ.

Under primed conditions, there was a 4-log increase in NO expression with TNF-α priming and 6-log increase with IFN-γ priming when compared to the combined TNF-α and IFN-γ activation respectively. Although these are theoretical results and our model does not include an NO depletion term, multiple *in vitro* experiments have shown a significant increase in the levels of NO following cytokine priming when compared to LPS controls. The increase in NO should presumably enable primed cells to more readily eliminate bacterial pathogens [26, 27, 89, 90]. In addition, the increased NO production potential of IFN-γ primed macrophages located in the periphery of the inflammatory microenvironment may help reduce bacterial dissemination to adjacent cells and tissues [91, 92].

**Fig 9:**
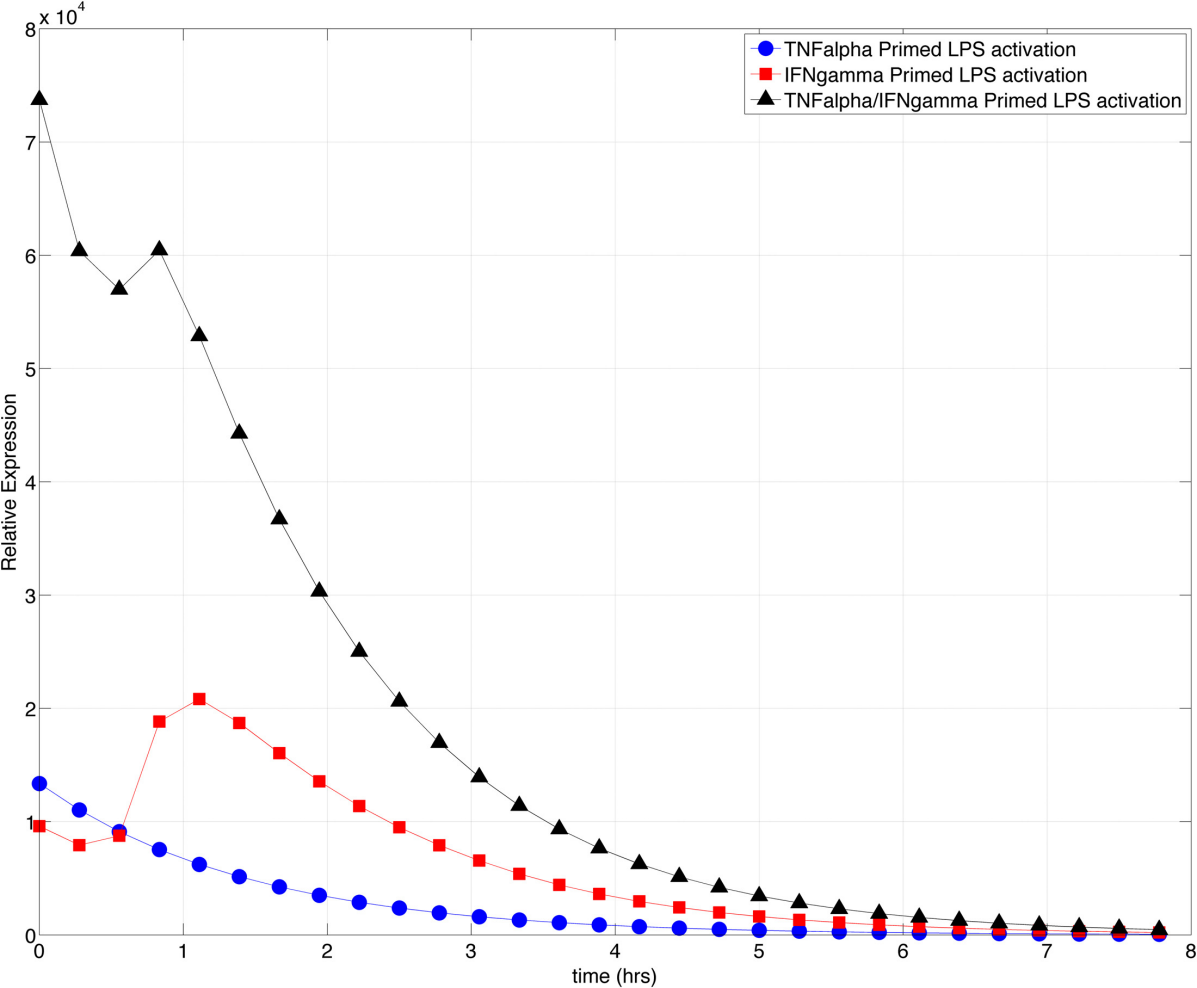
TNF-α and IFN-γ primed iNOS Gene Expression Dynamics. The model was simulated with TNF-α, IFN-γ, or both TNF-α /IFN-γ for 24 simulation hours to prime the system. The concentrations of iNOS transcription factors, iNOS mRNA, and iNOS protein were used as inputs to the system for an 8-hour addition simulation under LPS activation. The resulting dynamics display the behavior of iNOS gene expression under primed conditions. The combination of both, TNF-α and IFN-γ causes a significant increase in the dynamics of the primed system however, IFN-γ priming has the ability to generate an increase in the proinflammatory burst when compared to the initial value of iNOS gene expression after IFN-γ priming.

The highest oxidative burst is seen when both, IFN-γ and TNF-y are used to prime the system. The synergy of both cytokines induces a 7-log increase in levels of NO production when compared to non-primed stimulation by both cytokines. The levels of iNOS protein are also greater when primed with both cytokines as opposed to when activated with both cytokines. Similar to the non-primed condition, under priming, although TNF-α can induce a faster NO production rate, IFN-γ has the capacity to activate the expression of additional TNF-α and further increase the expression levels of both iNOS protein and NO (Fig 9). The dynamics of the TNF-α and IFN-γ pathway intermediates can be found in S2–S4 Figs.

Empirical observations suggest that the phenomenon of priming enables macrophages to produce an immediate proinflammatory response against pathogenic infections [93]. We plotted the temporal iNOS gene expression under primed conditions to determine the magnitude and response dynamics of the primed immune system. Previously we observed in cytokine-activated models that TNF-α induced a faster iNOS expression whereas IFN-γ induced a delayed but higher magnitude of iNOS expression (Figs 5F and 6F). In priming however, both cytokines induced an initial level of iNOS gene expression with TNF-α priming resulting in a higher initial concentration. However, upon subsequent LPS stimulation post priming, the magnitude of iNOS gene expression is less than the initial concentration generated by priming, exhibiting an exponential decay in the levels of iNOS over 8 hours (Fig 9).

IFN-γ however, has a lower starting iNOS concentration post-priming but induces a higher magnitude of iNOS gene expression at 1-hour post LPS stimulation. These results suggest, comparatively, that the iNOS effector response is short lived during TNF-α priming but relatively sustained during IFN-γ priming. According to the architecture of the microenvironment of the infected cell (Fig 1), TNF-α priming would occur mainly in the second condition, that is, macrophages adjacent to the primary site of infection. TNF-α priming would prove beneficial in this region as some bacteria that overcome the host defenses in the initial, non-primed resting macrophages could potentially be rapidly eliminated by the adjacent macrophages. IFN-γ priming would similarly occur primarily in condition four, macrophages on the periphery of the infection, and therefore, the delayed iNOS expression time may become beneficial for macrophages located in this region as the increase in magnitude proceeds the decay in iNOS gene expression within TNF-α primed macrophages and ultimately, enables cells on the periphery to extend the duration of the proinflammatory response.

The combined priming by both cytokines increases the initial concentration of iNOS mRNA approximately seven times greater than individual priming levels. The dynamics of priming with both cytokines highlights the synergism of IFN-γ and TNF-α in the proinflammatory response enabling the macrophage to have a large initial oxidative burst followed by a subsequent increase in iNOS, which not only extends the effector response but increases the magnitude of the response [94].

**Fig 10:**
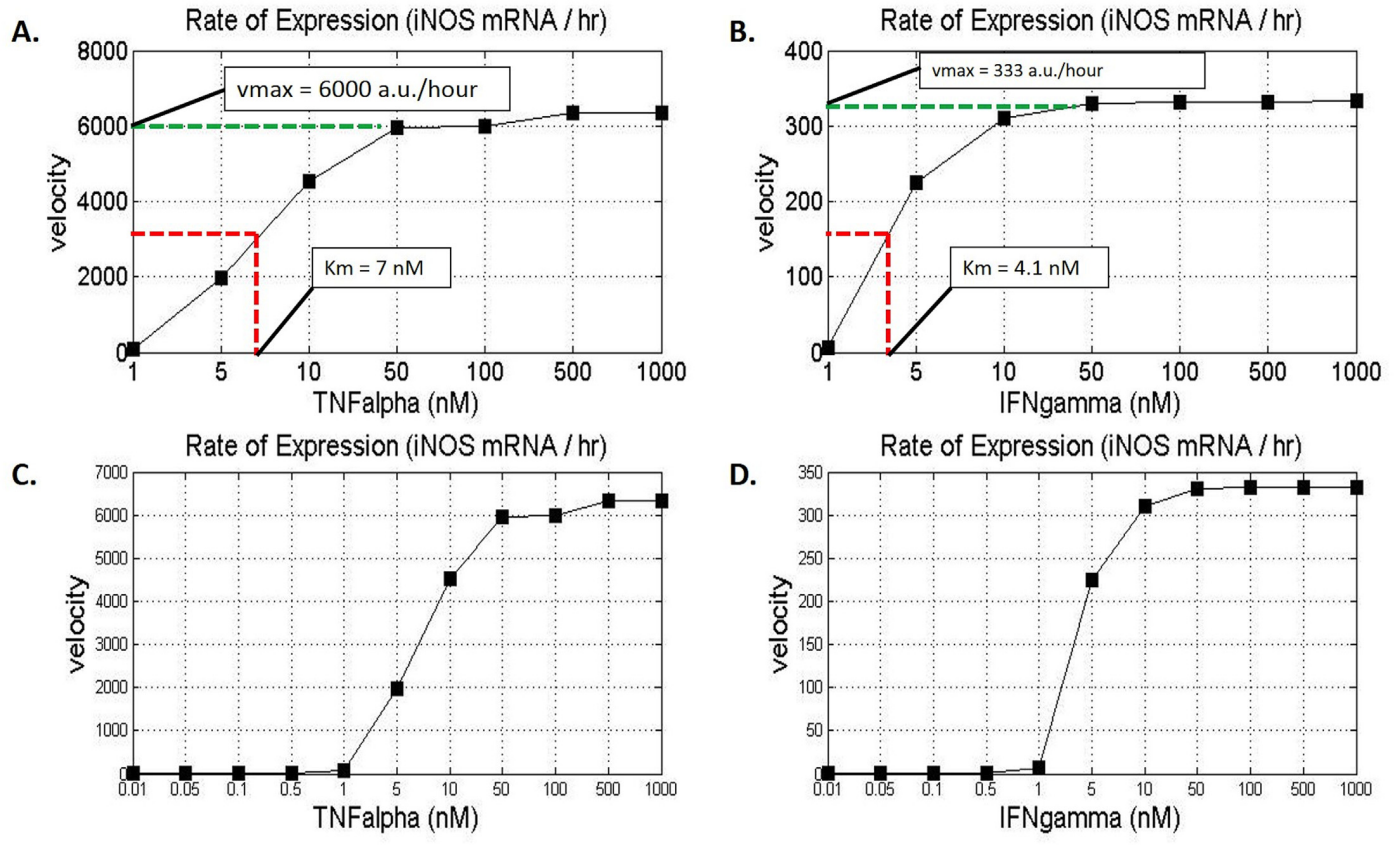
iNOS Gene Expression as a Function of TNF-α or IFN-γ. A range of concentrations of TNF-α and IFN-γ were used to stimulate the system and the maximum iNOS gene expression rates were plots as a function of cytokine concentration. The time to reach maximal expression under varying concentrations of cytokines was used to derive the velocity of expression under each respective condition. (A) The Michaelis-Menten type plot shows the rate of iNOS gene expression as a function of TNF-α. Since TNF-α induces peak iNOS expression faster than IFN-γ, the rate of expression is greater for TNF-α than for IFN-γ. (B) The Michaelis-Menten type plot shows the rate of iNOS gene expression as a function of IFN-γ. The Km value is marked at half-maximal velocity. (C) The expanded graph of TNF-α dependent iNOS gene expression rate shows a sigmoidal curve suggesting a cytokine threshold for gene activation. (D) The expanded graph of IFN-γ dependent iNOS gene expression rate also shows a sigmoidal curve in addition to a smaller range upon which Vmax is reached. Vmax values are in arbitrary units, which correlate to the change of relative expression per time.

Since both, TNF-α and IFN-γ have distinct rates of iNOS gene activation, we sought to quantify the rate of iNOS gene expression as a function of TNF-α or IFN-γ concentration. The peak iNOS mRNA concentration values for specific cytokine concentrations were captured and divided by the time the peak value was reached to determine the iNOS production velocity for the given cytokine concentration. The resulting iNOS mRNA production velocity was determined and plotted for a range of TNF-α and IFN-γ concentrations (Fig 10). Earlier we primed the system with 1 nM IFN-γ and therefore, we chose an initial value of 1 nM for both cytokines and incremented the cytokine concentration. Although literature has suggested that under systemic pathogenic infection, the peak IFN-γ concentrations reached about 3.6 nM and TNF-α reached a concentration that was about 6 times lower, we increased our cytokine concentration to a theoretical saturation level of 1000 nM to capture a full range of dynamics [95–97].

We found that both cytokines increased the rate of iNOS gene expression to a steady-state level representing a pseudo-Michaelis-Menten type enzyme kinetic relationship (Fig 10A and 10B). Generally the steady-state Michaelis-Menten plots graphically display the maximum velocity (Vmax) of an enzyme with respect to a substrate concentration, and the graph can be used to determine the Michaelis-Menten constant (Km) which is the substrate concentration at which half-maximal velocity is reached (39). Similarly, we used our plots to determine the Km and Vmax values of iNOS gene expression for the two-proinflammatory cytokines (Fig 10A and 10B). The Vmax for TNF-α activated iNOS gene expression was at a steady state level of 6000 arbitrary units (a.u.) per hour with a Km of 7 nM. The Vmax for IFN-γ activated iNOS gene expression was lower at a steady state level of about 333 a.u. per hour and a Km of 4.1 nM. Although the Km for TNF-α is seemingly higher than that of IFN-γ, the velocity of iNOS gene expression for 4.1 nM TNF-α, which is the Km of IFN-γ, is more than ten times the iNOS gene expression velocity generated by the same concentration of IFN-γ. This suggests that the system is more sensitive to lower concentrations of TNF-α than lower concentrations of IFN-γ in terms of iNOS gene expression. The difference in sensitivity to the system by these cytokines supports the earlier time-to-peak response time of iNOS gene expression observed under TNF-α stimulation. In addition, these observed kinetics also justify the need for a higher priming and activation concentration of IFN-γ as opposed to TNF-α.

*In vivo* experiments by Damas et al a have suggested that the TNF-α receptor (TR1) is a high affinity receptor since the concentration of TNF-α in bodily fluids under pathophysiological conditions such as systemic sepsis is usually very low (in the picomolar range) [98]. Subsequent *in vitro* experiments by Grell et al strengthened the claim by calculating the high affinity TR1 dissociation constant to be 0.02 nM [64]. Contrastingly, Loon et al and Taniguchi et al showed how the IFN-γ receptors are also high affinity receptors with a dissociation constant ranging from 0.01 nM to 0.02 nM [99, 100]. We therefore expanded our cytokine activation range to include concentrations as low as 0.01 nM to compliment the dissociation constants of the two receptors in order to derive insight on the behavior of iNOS gene expression at lower cytokine concentrations, which we used to generate a representation of the dynamics of iNOS expression for cytokine priming at lower concentrations of TNF-α and IFN-γ (Fig 10C and 10D).

Both plots resulted in a sigmoidal curve suggesting a cytokine activation threshold. While TNF-α showed a marked increase between 1 nM and 50 nM, IFN-γ showed a marked increase only between 1–10 nM. Although TNF-α activation resulted in a higher rate of iNOS gene expression, our previous results on iNOS gene expression dynamics prove that this does not necessarily correlate to the magnitude of gene expression as IFN-γ clearly induced a larger concentration of iNOS mRNA (Fig 6F). Rather, it likely correlates to the earlier time to peak activation observed under TNF-α stimulation (Fig 8). Therefore, the smaller activation interval of IFN-γ may correlate with the strength of cytokine activation and IFN-γ’s ability to saturate the system at lower concentrations than with TNF-α.

The plots above also highlight the benefit of priming at lower concentrations since priming with large concentrations of either cytokine has been known to be more detrimental than beneficial. For example, Hu et al described the advantage of using a lower dosage of IFN-γ for priming. They showed that under a low dosage of IFN-γ priming, STAT1 levels increase to a high steady state level and the inhibitory SOCS1 protein is short lived whereas under a high dosage of IFN-γ, both STAT1 and SOCS1 linearly increase over a period of 24 hours. The continuous increase of the proinflammatory STAT1 protein causes greater stress on the macrophage by producing non-gradual oxidative bursts while the increase in SOCS1 can cause an equally strong anti-inflammatory response that are ultimately detrimental to both the pathogen and the cell [87, 101]. Therefore, priming at a TNF-α concentration of 0.05 and an IFN-γ concentration of 1 nM not only correlate to comparable iNOS gene expression velocities, but may minimally activate anti-inflammatory molecules while generating a pool of proinflammatory species ready to combat disseminating pathogenic infections.

## Conclusions

We developed a comprehensive kinetic model that captures the gene expression dynamics of induced nitric oxide synthase, an essential enzyme utilized by macrophages in the proinflammatory response against intracellular pathogens. Our model was used to simulate the host-effector response under various stimulatory conditions and ultimately, under cytokine priming conditions. We focused on two key inflammatory cytokines, TNF-α and IFN-γ, and used LPS in the system to represent varying levels of intracellular pathogens.

Our model codifies elemental interactions and respective reactive rates that impact host proinflammatory response, however, the *in silico* study we performed using the model provided insight into the relative role and synergistic contribution of TNF-α and IFN-γ in NO actuation. Moreover, we used our model to recreate the dynamics of the proinflammatory response to these cytokines and to further comparatively investigate the effects of various cytokine environments prior to and during infection (Fig 1). In so doing, we observed that TNF-α clearly has a significant impact temporally on when the initial peak iNOS expression occurs, while IFN-γ mainly impacts the maximum magnitude of expression. These new observations were not directly coded into the model. Rather, similar to observations from *in vitro* systems tested under respectively similar conditions, our observations are the combined outcome of the inputs to the *in silico* model and the outputs resulting from the model.

**Fig 11:**
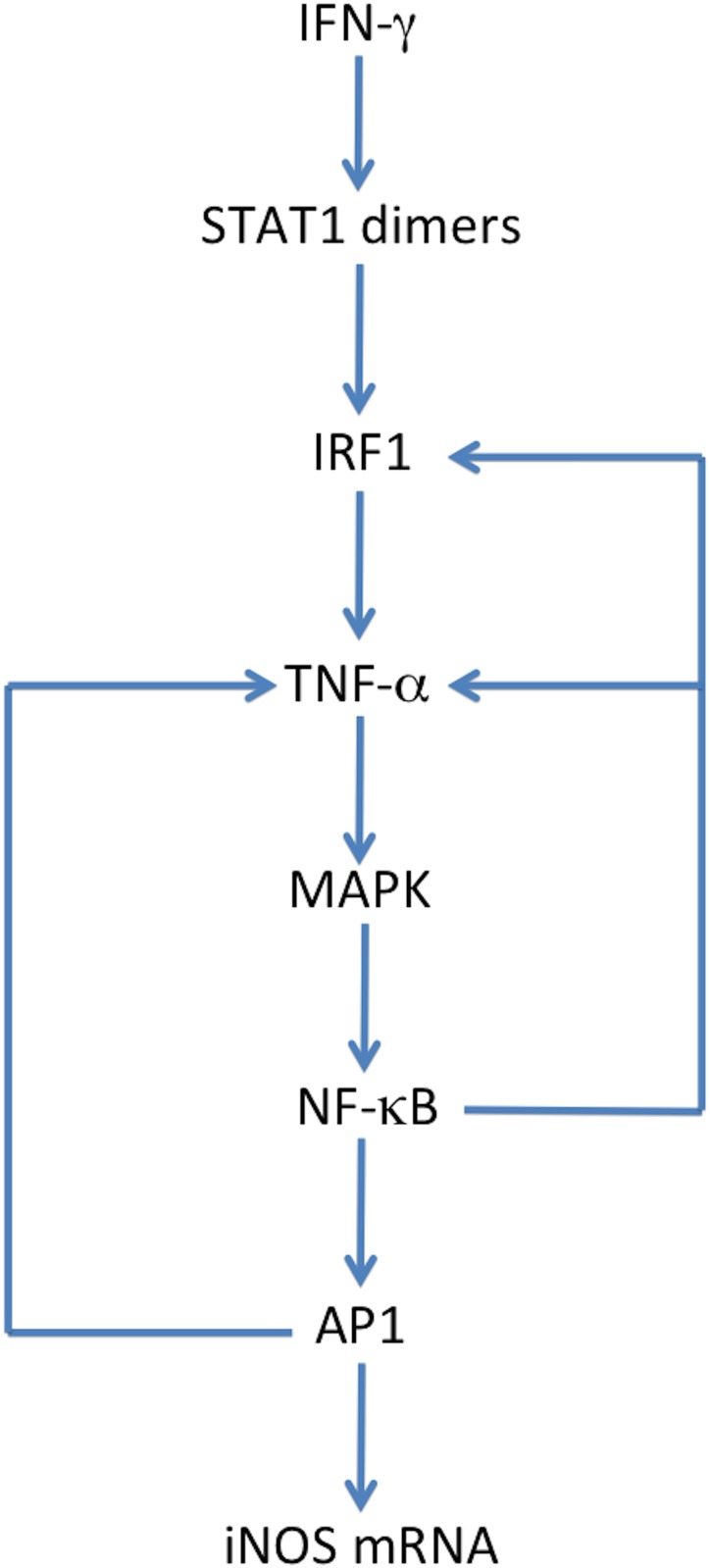
Mechanism of IFN-γ Priming. The consolidated signal transduction system represented here shows how IFN-γ priming directly stimulates the system to ultimately generate iNOS.

Our model also helped clarify the mechanistic importance of IFN-γ priming for pathogen clearance in macrophages. Specifically, similar to experimental studies that have shown that IFN-γ priming is essential for a more robust proinflammatory response, IFN-γ priming in our model exhibited the highest production of iNOS and NO when compared to non-priming conditions. Although TNF-α priming generates a more rapid and robust response than without priming, only under IFN-γ priming do we see an increase in the maximum iNOS expression upon subsequent stimulation with LPS. The mechanistic explanation for the observed effects of IFN-γ priming can be seen in the consolidated system represented in Fig 11. IFN-γ is the only activator of STAT1 phosphorylated dimers within our system. The activation of STAT1 phosphorylated dimers can then lead to an increase in the amount of IRF1 which has the ability to express TNF-α mRNA [34]. The IRF1 mediated production of TNF-α mRNA is the postulated bridge between IFN-γ and iNOS since TNF-α induces NF-κB and AP1, the remaining transcription factors needed for iNOS mRNA. Thus, our model provides an explanatory mechanism that demonstrates the ability of IFN-γ to express iNOS in the absence of LPS. Furthermore, both AP1 and NF-κB induce a feed-forward loop by activating more TNF-α and IRF1 gene expression respectively. The culmination of these interactions results in iNOS gene expression and ultimately, nitric oxide formation. Thus, a small amount of IFN-γ is needed to initiate the two feed forward loops, which then increase the amount of iNOS gene expression exponentially, ultimately creating a “molecular ripple effect”.

Although there is room for expanding our current model, such as the addition of STAT1 gene expression, interleukins, and the consequential induction of anti-inflammatory cytokines to regulate the overall response, the model we developed provides significant insight on the importance of the mechanistic role and modulation of the proinflammatory response by TNF-α and IFN-γ. Ultimately, we anticipate that the synergistic effects and differential dynamics of proinflammatory cytokines quantified in our *in silico* study can be used to determine important immunomodulatory approaches for computational drug design against intracellular pathogens.

## Supporting Information

S1 Fig iNOS Gene Expression Optimization. The experimental results published by Mustafa et al were normalized and plotted against the simulated control expression of iNOS mRNA under LPS stimulation (23). The parameters were then optimized using freely available software, DAKOTA, which was created by Sandia National Labs. Sets 1, 2, and 3 represent the three best-fitted set of parameters to the experimental results.
(TIFF)

S2 Fig MAPK Intermediates. The MAPK intermediates plotted here represent the activation propagation from LPS activated complex to upper and lower MAPK pathways and their regulatory phosphatases, MKP1 and MKP5. IFN-y priming condition was simulated for 24 hours upon which the end values of the priming were used as initial conditions for LPS and IFN-y activation condition that was simulated for 8 hours.
(PDF)

S3 Fig NF-κB Pathway Intermediates. The NFkB intermediates plotted here represent the activation propagation IKK to the dissociation of the IkBa-NFkB cytoplasmic complex and eventual IkBa-mRNAn expression. The rate equations and parameters were initially taken from the model published by Sharp et al (10) and the units were modified accordingly to fit our model.
(PNG)

S4 Fig Arginine-Citrulline Cycle. The three species plotted here represent the simulation of the arginine-citrulline cycle. Through the action of arginosuccinate synthase and arginosuccinate lyase, arginine is replenished back into the system after it’s utilization by iNOS to produce NO.
(PNG)

S1 File Supplementary Data File. Single data file containing all supplementary tables and figures, and additional information on model development and implementation.
(PDF)

S1 Table Model Reactions and Parameters. (PDF)

S2 Table Initial Conditions of All Reaction Species within Model. (PDF)
