# Supplementary figures and images for: Investigating the Role of TNF-α and IFN-γ Activation on the Dynamics of iNOS Gene Expression in LPS Stimulated Macrophages
Source: PLoS One. 2016 Jun 8;11(6):e0153289. doi: 10.1371/journal.pone.0153289 (PMC4898755; doi:10.1371/journal.pone.0153289)

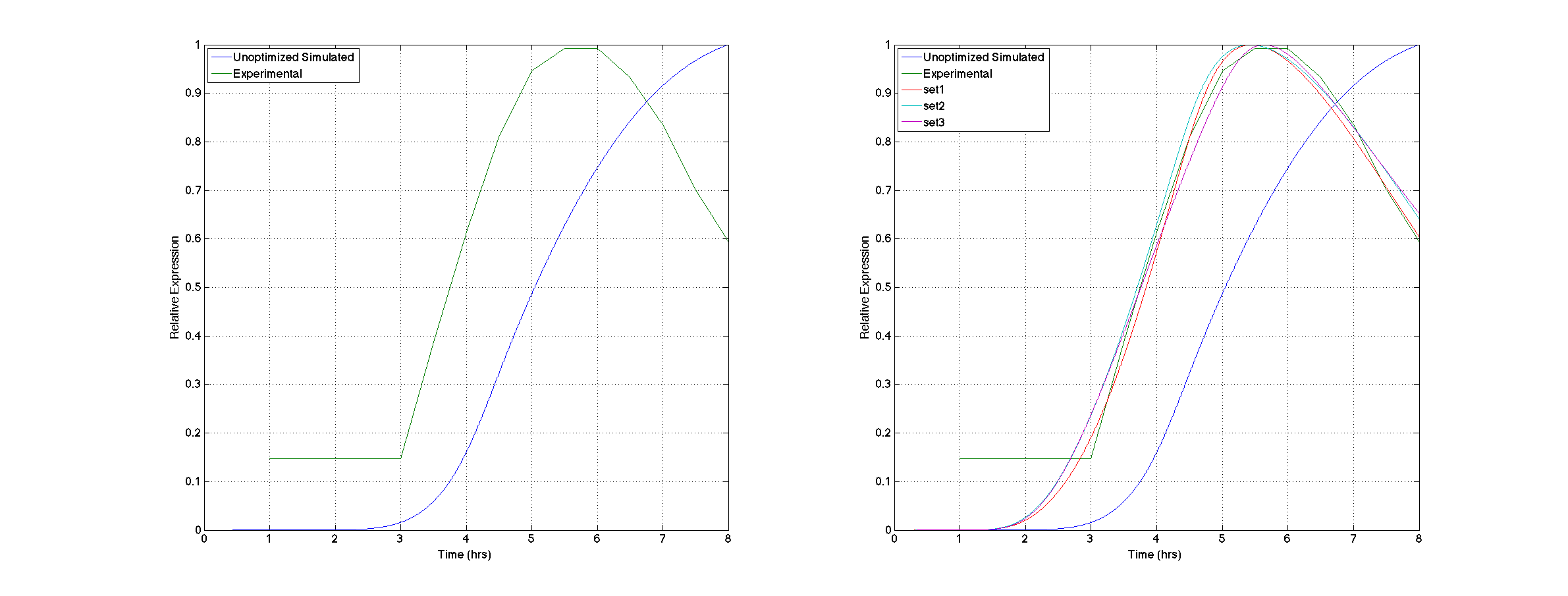

Supplement: S1 Fig — The experimental results published by Mustafa et al were normalized and plotted against the simulated control expression of iNOS mRNA under LPS stimulation (23). The parameters were then optimized using freely available software, DAKOTA, which was created by Sandia National Labs. Sets 1, 2, and 3 represent the three best-fitted set of parameters to the experimental results. (TIFF) [file pone.0153289.s001.tiff]

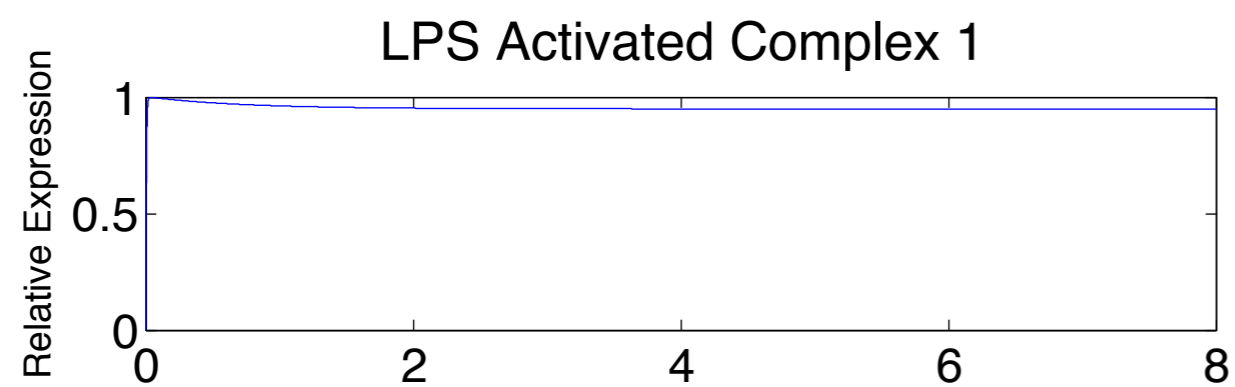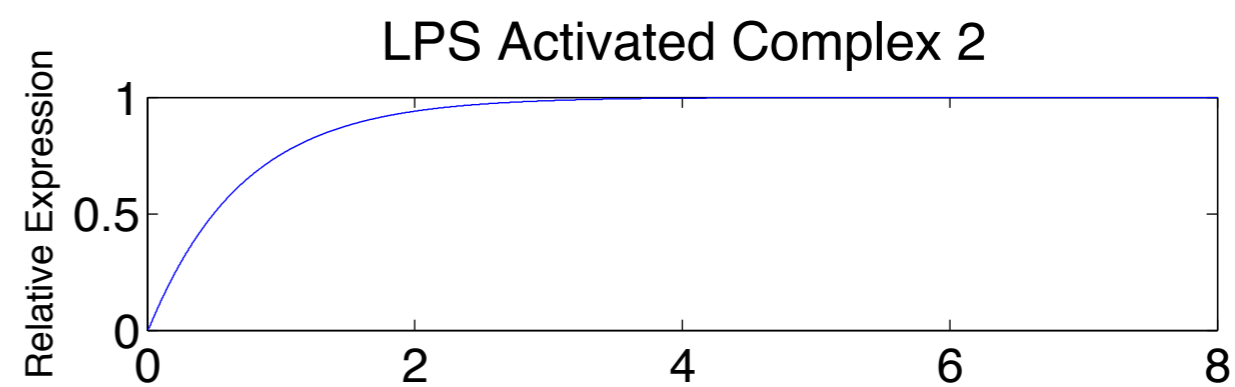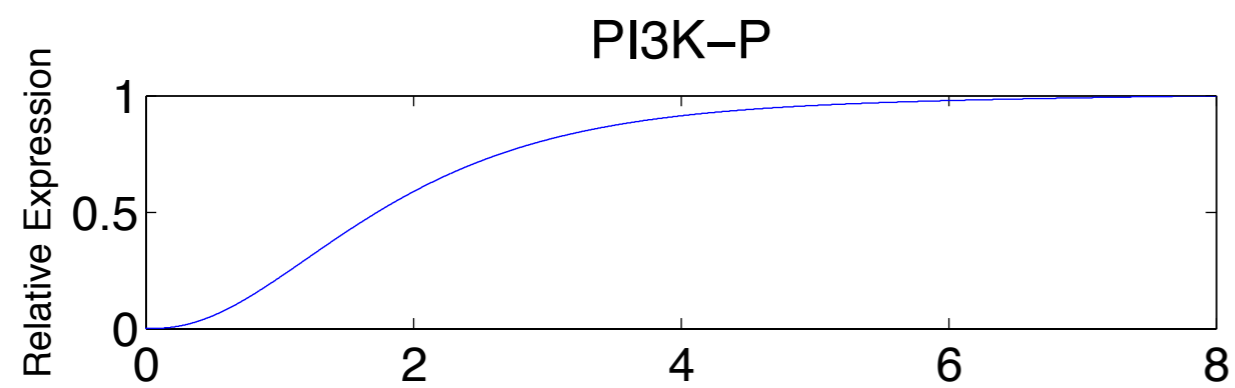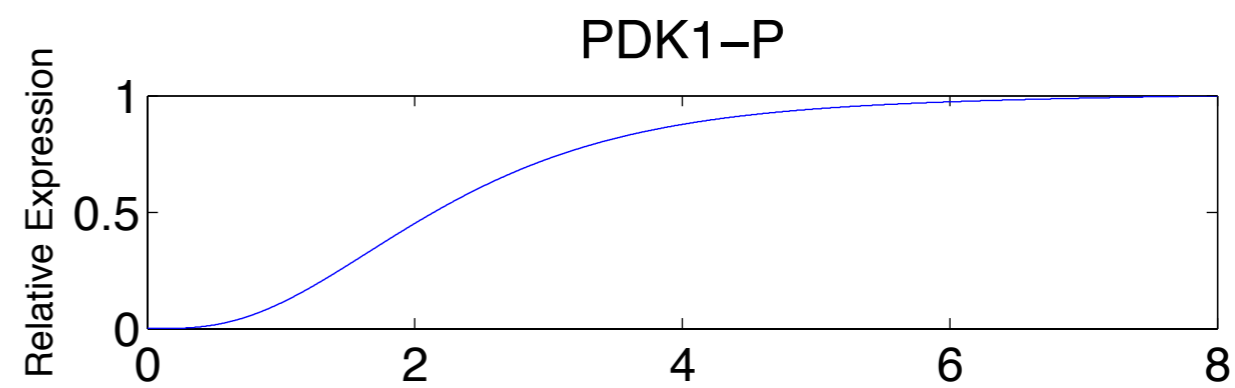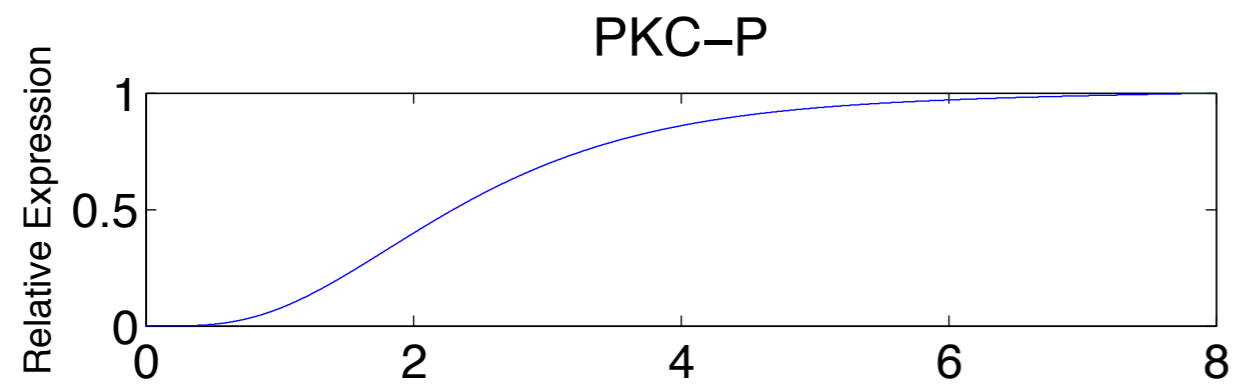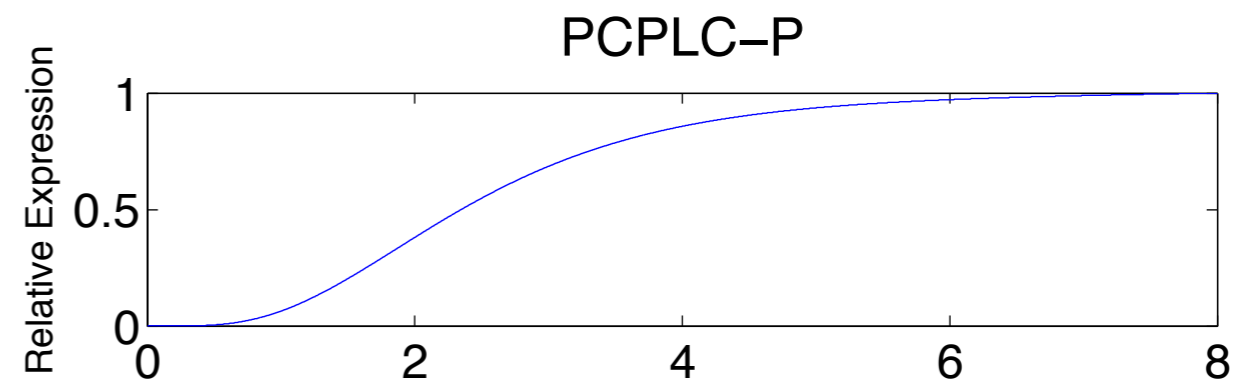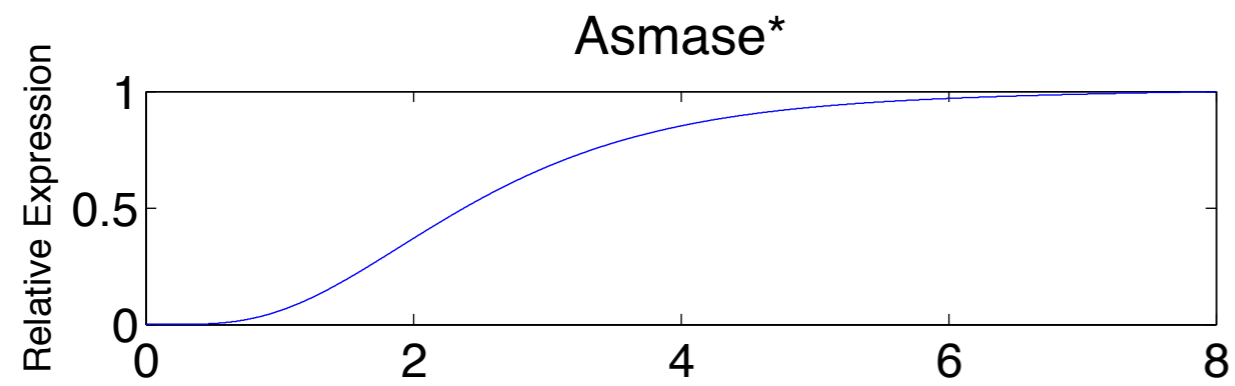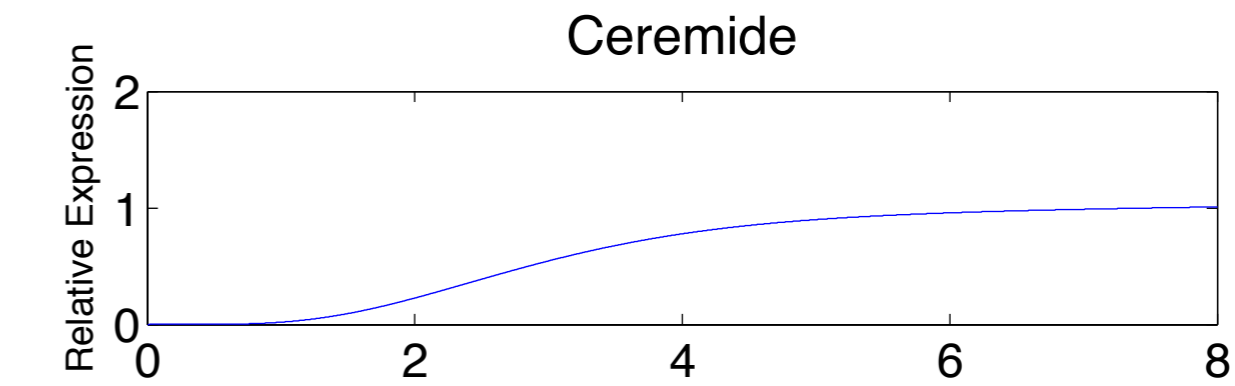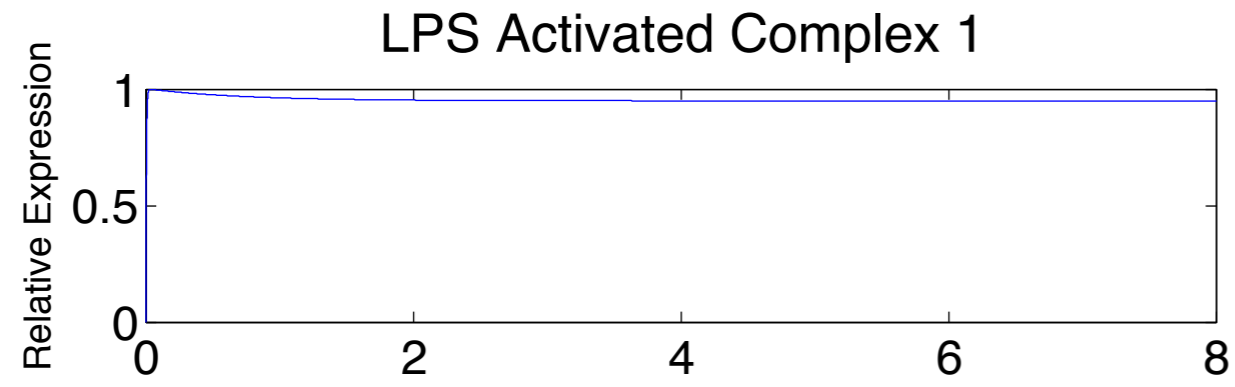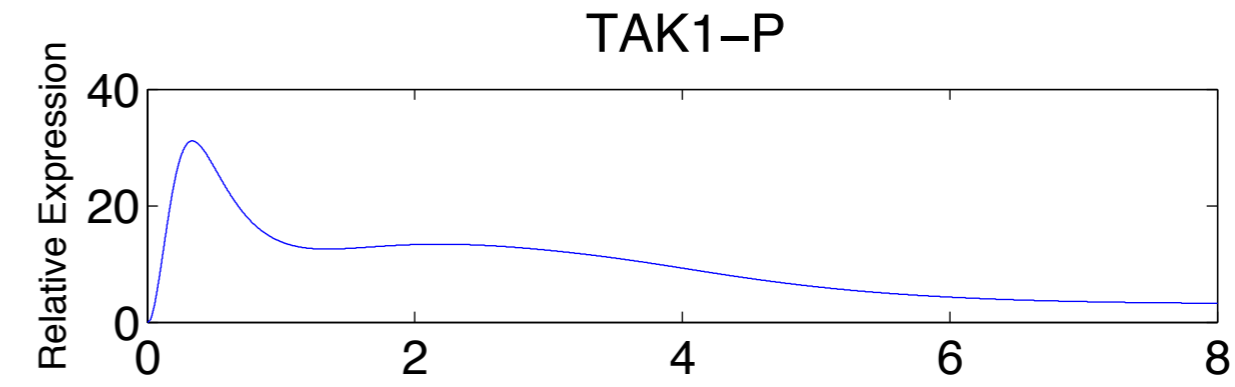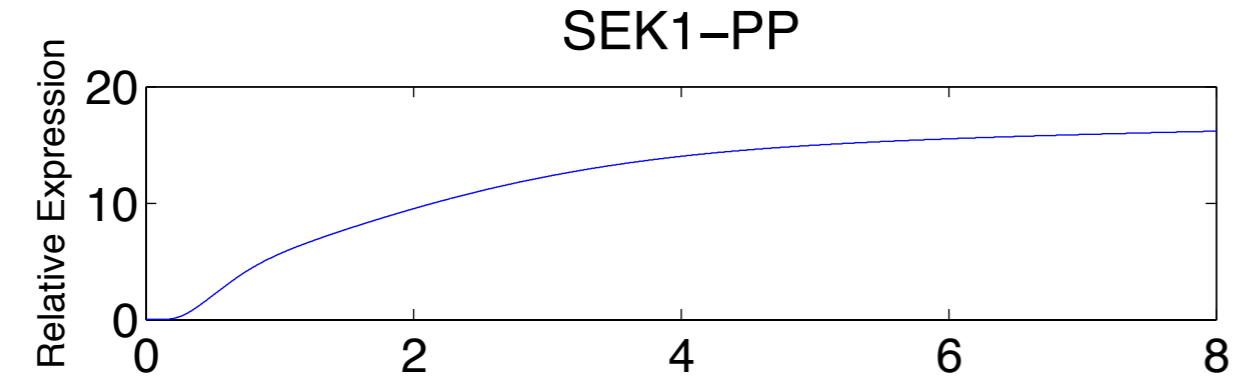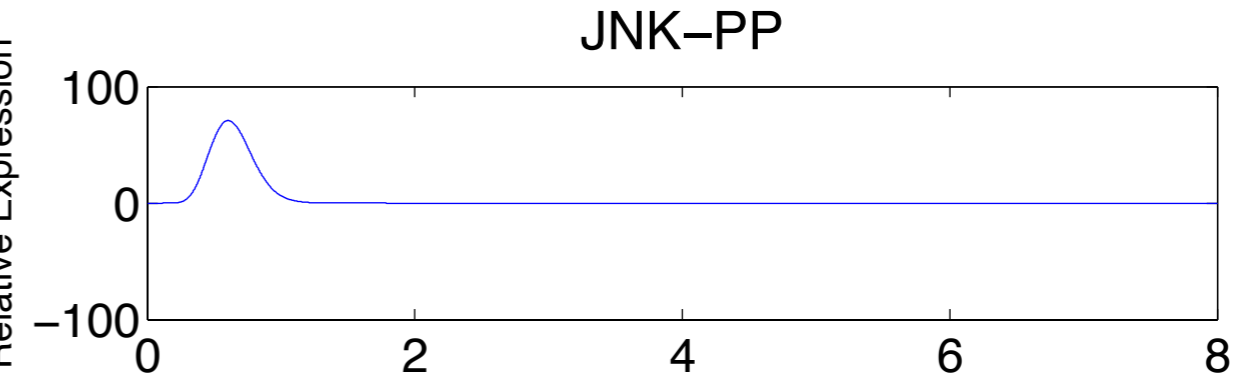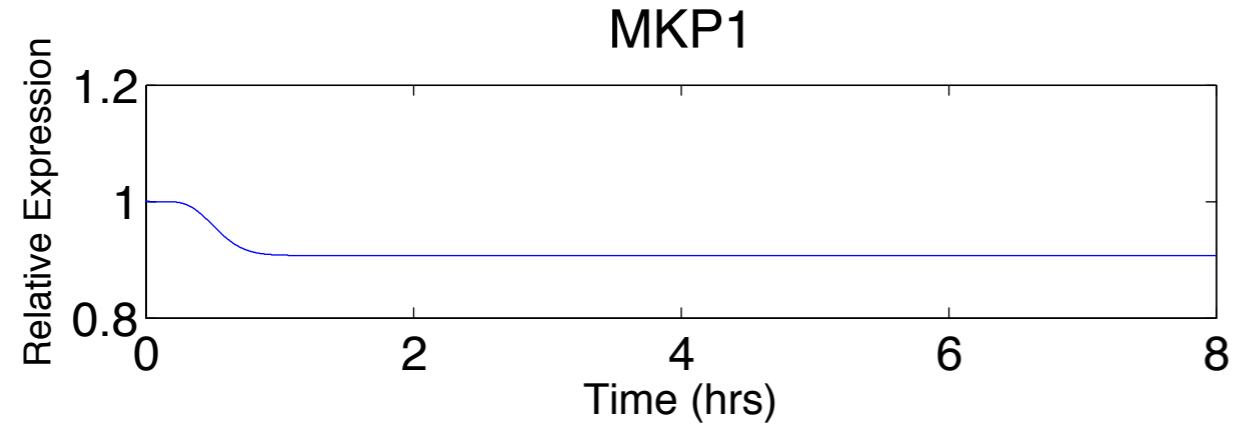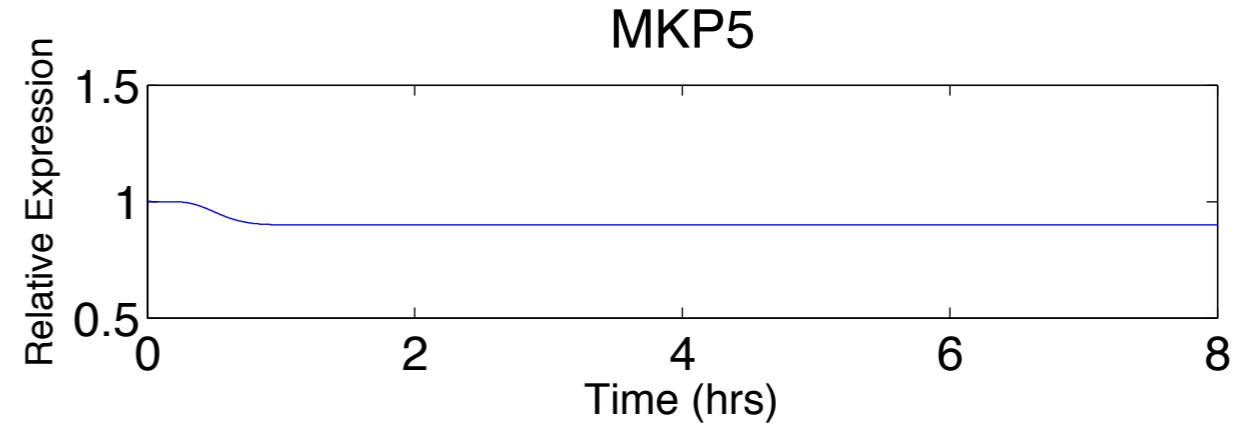

Supplement: S2 Fig — The MAPK intermediates plotted here represent the activation propagation from LPS activated complex to upper and lower MAPK pathways and their regulatory phosphatases, MKP1 and MKP5. IFN-y priming condition was simulated for 24 hours upon which the end values of the priming were used as initial conditions for LPS and IFN-y activation condition that was simulated for 8 hours. (PDF) [file pone.0153289.s002.pdf]

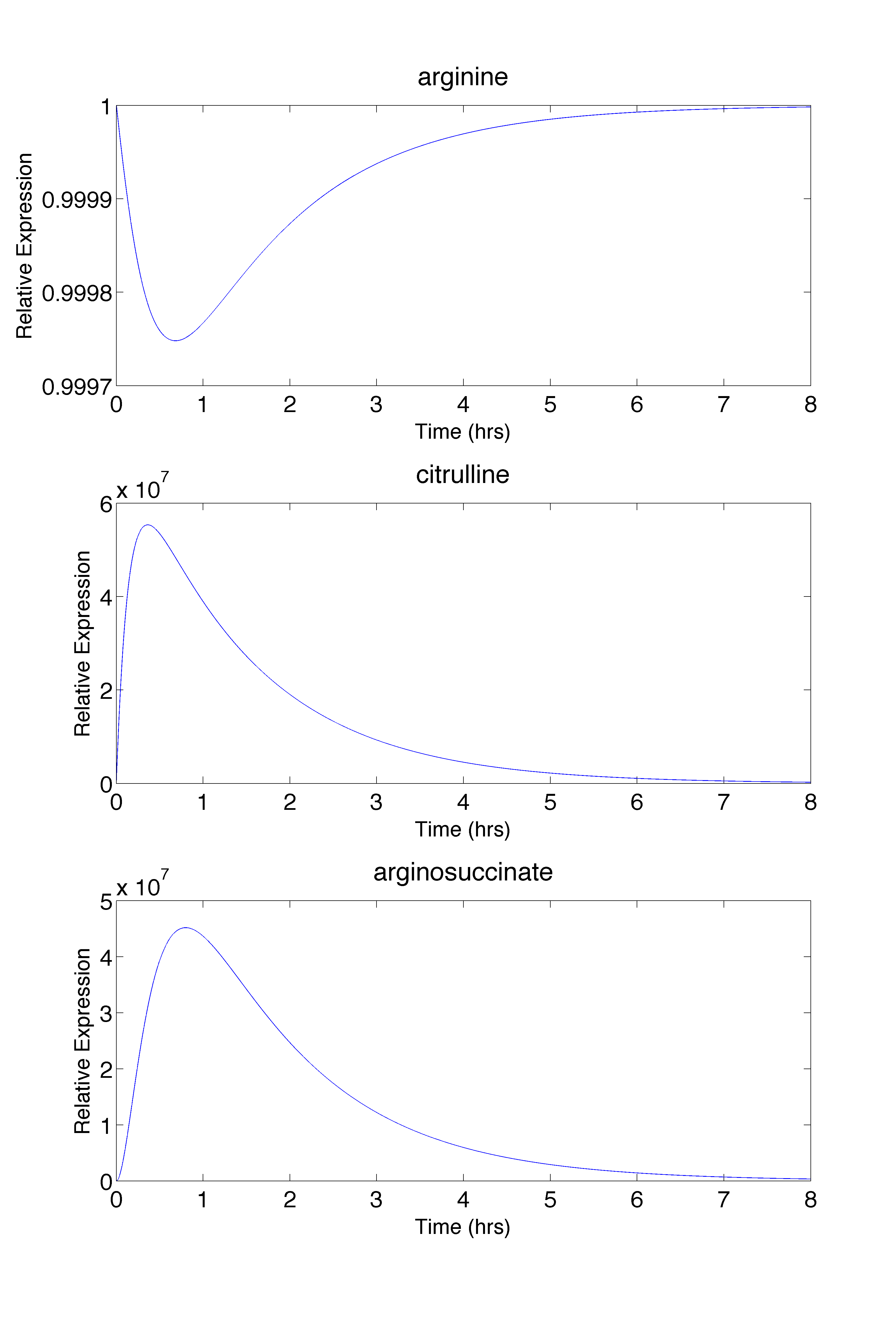

Supplement: S4 Fig — The three species plotted here represent the simulation of the arginine-citrulline cycle. Through the action of arginosuccinate synthase and arginosuccinate lyase, arginine is replenished back into the system after it’s utilization by iNOS to produce NO. (PNG) [file pone.0153289.s004.png]
