# Supplementary material for: Investigating the Role of TNF-α and IFN-γ Activation on the Dynamics of iNOS Gene Expression in LPS Stimulated Macrophages
Source: PLoS One. 2016 Jun 8;11(6):e0153289. doi: 10.1371/journal.pone.0153289 (PMC4898755; doi:10.1371/journal.pone.0153289)
Supplement: S1 File — Single data file containing all supplementary tables and figures, and additional information on model development and implementation. (PDF) [file pone.0153289.s005.pdf]

Investigating the Role of TNF-α and IFN-γ Activation on the Dynamics of iNOS Gene Expression in LPS Stimulated Macrophages

Supplementary Data

Taha Salim, Cheryl L. Sershen, Elebeoba E. May

The JAK/STAT pathway reactions and parameters are not inclusive within this text since it has been published by Yamada et al (1). Our model integrates the Yamada model with the addition of compartmentalization.

Supplementary Table 1: Model Reactions and Parameters

Reaction	Function	Rate Constant	Parameters	Units	References
**[LPS]+[LBP] → [LPS-LBP]**	Binding	k77_1	2.5	$nM^{-1}s^{-1}$	(2)
**[LPS-LBP] → [LPS] + [LBP]**	Dissociation	k77_1minus	0.0251	s^{-1}	(2)
**[LPS]+[CD14] → [LPS-CD14]**	Receptor binding	k77_2	2.86e-6	$nM^{-1}s^{-1}$	(2)
**[LPS-CD14] → [LPS] + [CD14]**	Dissociation	k77_2minus	0.0251	s^{-1}	(2)
**[LPS-LBP] + [CD14] → [LPS-CD14] + [LBP]**	LPS Transfer	k77_3	0.00286	$nM^{-1}s^{-1}$	(2)
**[LPS-CD14] → [LPS] + [CD14]**	Dissociation	k77_3minus	0.0251	s^{-1}	(2)
**[TLR4]+[MD2] → [TLR4-MD2]**	Helper protein binding	k77_4	6.47e-6	$nM^{-1}s^{-1}$	(2)
**[TLR4-MD2] → [TLR4] + [MD2]**	Dissociation	k77_4minus	3.65e-4	s^{-1}	(2)
**[LPS-CD14]+[TLR4] → [AC1]**	Membrane complex 1 formation	k77_5	2.33e-6	$nM^{-1}s^{-1}$	(2)
**[AC1] → [LPS-CD14]+[TLR4]**	Dissociation	k77_5minus	6.34e-2	s^{-1}	(2)
**[LPS-CD14]+[TLR4-MD2] → [AC2]**	Membrane complex 2 formation	k77_6	4.37e-4	$nM^{-1}s^{-1}$	(2)
**[AC2] → [LPS-CD14]+[TLR4-MD2]**	Dissociation	k77_6minus	4.03e-2	s^{-1}	(2)
**[AC1]+[PI3K] → [AC-PI3K]**	Binding Reaction	k79	4.80e-4	$nM^{-1}s^{-1}$	(3)
**[AC2]+[PI3K] → [AC-PI3K]**	Binding Reaction	k79	4.80e-4	$nM^{-1}s^{-1}$	(3)
**[AC-PI3K] → sink**	Complex Degradation	k80	2.81e-5	s^{-1}	(3)
**[AC-PI3K] → [PI3K_P]**	PI3K Phosphorylation	k81	2.82e-4	s^{-1}	(3)

**[PI3K_P]+[PDK1] → [PI3K-PDK1]**	Binding Reaction	k82	7.56e-5	$nM^{-1}s^{-1}$	(3)
**[PI3K_P-PDK1] → sink**	Complex Degradation	k83	1.05e-5	s^{-1}	(3)
**[PI3K_P-PDK1] → [PDK1_P]**	PDK1 Phosphorylation	k84	6.84e-4	s^{-1}	(3)
**[PDK1_P]+[PKC] → [PDK1_P-PKC]**	Binding Reaction	k85	0.016	$nM^{-1}s^{-1}$	(3)
**[PDK1_P-PKC] → sink**	Complex Degradation	k86	7.80e-8	s^{-1}	(3)
**[PDK1_P-PKC] → [PKC_P]**	PKC Phosphorylation	k87	0.016	s^{-1}	(3)
**[PKC_P]+[PCPLC] → [PKC_P-PCPLC]**	Binding Reaction	k88	2.08e-4	$nM^{-1}s^{-1}$	(4)
**[PKC_P-PCPLC] → sink**	Complex Degradation	k89	6.33e-4	s^{-1}	(4)
**[PKC_P-PCPLC] → [PCPLC_P]**	PCPLC_P Phosphorylation	k90	0.009	s^{-1}	(4)
**[PCPLC_P]+[Asmase] → [PCPLC_P-Asmase]**	Binding Reaction	k91	1.83e-4	$nM^{-1}s^{-1}$	(4)
**[PCPLC_P-Asmase] → sink**	Complex Degradation	k92	6.33e-4	s^{-1}	(4)
**[PCPLC_P-Asmase] → [Asmase*]**	Asmase Activation	k93	0.009	s^{-1}	(4)
**[Asmase*] + [sphingomyelin] → [ceremide]**	Enzymatic Reaction	k94<br>k95	103.83<br>590	nM/s<br>nM	(5)<br>(5)
**[ceremide]+[TAK1] → [ceremide-TAK1]**	Binding Reaction	k96	3.30e-4	$nM^{-1}s^{-1}$	(6)
**[ceremide-TAK1] → sink**	Complex Degradation	k97	2.17e-3	s^{-1}	(6)
**[ceremide-TAK1] → [TAK1_P]**	TAK1 Phosphorylation	k98	7.40e-3	s^{-1}	(6)
**[TAK1_P]+[SEK1] → [TAK1_P-SEK1]**	Binding Reaction	k99	2.57e-4	$nM^{-1}s^{-1}$	(7)
**[TAK1_P-SEK1] → sink**	Complex Degradation	k100	1.60e-5	s^{-1}	(7)
**[TAK1_P-SEK1] → [SEK1_P]**	SEK1 Phosphorylation	k101	5.64e-3	s^{-1}	(7)
**[TAK1_P]+[SEK1_P] → [TAK1_P-SEK1_P]**	Binding Reaction	k102	2.70e-3	$nM^{-1}s^{-1}$	(7)
**[TAK1_P-SEK1_P] → sink**	Complex Degradation	k103	1.60e-5	s^{-1}	(7)
**[TAK1_P-SEK1_P] → [SEK1_PP]**	SEK1_P Phosphorylation	k104	0.05	s^{-1}	(7)
**[SEK1_PP]+[JNK] → [SEK1_PP-JNK]**	Binding Reaction	k105	1.30e-3	$nM^{-1}s^{-1}$	(7)
**[SEK1_PP-JNK] → sink**	Complex	k106	1.60e-3	s^{-1}	(7)

	Degradation				
**[SEK1_PP-JNK] → [JNK_P]**	JNK Phosphorylation	k107	0.05	s^{-1}	(7)
**[SEK1_PP]+[JNK_P] → [SEK1_PP-JNK_P]**	Binding Reaction	k108	1.30e-2	$nM^{-1}s^{-1}$	(7)
**[SEK1_PP-JNK_P] → sink**	Complex Degradation	k109	1.60e-3	s^{-1}	(7)
**[SEK1_PP-JNK_P] → [JNK_PP]**	JNK_P Phosphorylation	k110	0.05	s^{-1}	(7)
**[JNK_PP] → [AP1]**	JNK_PP Nuclear Translocation	k111	3.40e-3	s^{-1}	(8)
**[AP1] → [JNK_PP]**	AP1 Cytoplasmic Translocation	k167	1.60e-4	s^{-1}	(8)
**[JNK_P]+[MKP1] → [JNK_P-MKP1]**	Binding Reaction	k112	0.01	$nM^{-1}s^{-1}$	(9)
**[JNK_P-MKP1] → sink**	Complex Degradation	k113	1	s^{-1}	(9)
**[JNK_P-MKP1] → [JNK]**	JNK_P Dephosphorylation	k114	0.05	s^{-1}	(9)
**[JNK_PP]+[MKP1] → [JNK_PP-MKP1]**	Binding Reaction	k115	0.045	$nM^{-1}s^{-1}$	(9)
**[JNK_PP-MKP1] → sink**	Complex Degradation	k116	1	s^{-1}	(9)
**[JNK_PP-MKP1] → [JNK_P]**	JNK_PP Dephosphorylation	k117	0.092	s^{-1}	(9)
**[JNK_P]+[MKP5] → [JNK_P-MKP5]**	Binding Reaction	k118	0.011	$nM^{-1}s^{-1}$	(9)
**[JNK_P-MKP5] → sink**	Complex Degradation	k119	0.99	s^{-1}	(9)
**[JNK_P-MKP5] → [JNK]**	JNK_P Dephosphorylation	k120	0.055	s^{-1}	(9)
**[JNK_PP]+[MKP5] → [JNK_PP-MKP5]**	Binding Reaction	k121	0.046	$nM^{-1}s^{-1}$	(9)
**[JNK_PP-MKP5] → sink**	Complex Degradation	k122	0.99	s^{-1}	(9)
**[JNK_PP-MKP5] → [JNK_P]**	JNK_PP Dephosphorylation	k123	0.093	s^{-1}	(9)
**[TAK1_P]+[IKK] → [TAK1_P-IKK]**	Binding Reaction	k124	8.93e-5	$nM^{-1}s^{-1}$	(10)
**[TAK1_P-IKK] → sink**	Complex Degradation	k125	1.0e-4	s^{-1}	(10)
**[TAK1_P-IKK] → [IKK_P]**	IKK Phosphorylation	k126	0.1	s^{-1}	(10)
**[IkBa]+[NFkBc] → [IkBa_NFkBc]**	NFkBc Deactivation	k127	0.5	$nM^{-1}s^{-1}$	(10)
**[IkBa_NFkBc] → sink**	Complex Degradation	k128	3.96e-4	s^{-1}	(10)

**[IKK_P]+[IkBa_NFkBc]→[IKK_P-IkBa-NFkBc]**	Binding Reaction	k129	0.185	$nM^{-1}s^{-1}$	(10)
**[IKK_P-IkBa_NFkBc] → sink**	Complex Degradation	k130	0.0125	s^{-1}	(10)
**[IKK_P-IkBa_NFkBc] → [IKK_P] + [NFkBc]**	NFkBc Activation	k131	0.0204	s^{-1}	(10)
**[IkBa_NFkBc] → [IkBa] + [NFkBc]**	Dissociation Reaction	k132	2.61e-5	s^{-1}	(10)
**[NFkBc] → [NFkBn]**	Nuclear transport of NFkB	k133	0.09	s^{-1}	(10)
**[NFkBn] → [NFkBc]**	Cytoplasmic transport of NFkB	k134	8.0e-5	s^{-1}	(10)
**$[NFkB]^2$ + [STAT1n_P_STAT1n_P] → [IRF1_mRNAn]**	IRF1 Gene Expression	k135<br>k135b<br>k136<br>k137<br>KIirf2	4.43e-3<br>0.03<br>1.94<br>10<br>364	nM/s<br>nM/s<br>nM<br>nM<br>nM	(1, 11, 12)
**[IRF1_mRNAn] → [IRF1_mRNAc]**	Cytoplasmic transport of mRNA	k138	1.36e-3	s^{-1}	(1)
**[IRF1_mRNAc] → [IRF1c]**	Translation	k139	0.01	s^{-1}	(1)
**[IRF1c] → [IRF1n]**	Nuclear Transport of IRF1	k140	5e-3	s^{-1}	(1)
**$[NFkB]^3$ + [AP1] → [TNFalpha_mRNAn]**	TNF-a Gene Expression	k141<br>k142<br>k143	0.01<br>3<br>4	nM/s<br>nM<br>nM	(11, 13, 14)
**$[IRF1n]^2$ + [IRF2n]→ [TNFalpha_mRNAn]**	TNF-a Gene Expression	k168<br>k169<br>KIirf2	1.38e-3<br>4.99e-3<br>364	nM/s<br>nM<br>nM	(15-17)
**[TNFalpha_mRNAn] → [TNFalpha_mRNAc]**	Cytoplasmic transport of mRNA	k144	8.05e-4	s^{-1}	(1)
**[TNFalpha_mRNAc] → [TNFalphac]**	Translation	k145	0.01	s^{-1}	(1)
**[TNFalphac] → [TNFalphaEC]**	Cellular Export of TNFa	k146	0.1	s^{-1}	(1)
**[TNFalphaEC] + [TR1] → [TNFR1]**	Receptor Binding	k147	0.0183	$nM^{-1}s^{-1}$	(18)
**[TNFR1] → [TNFalphaEC] + [TR1]**	Dissociation	k148	3.5e-4	s^{-1}	(18)
**[TNFR1] + [TRADD] ←→ [TNFR1_TRADD]**	Membrane complex formation	k151	0.1	$nM^{-1}s^{-1}$	(18)
**[TNFR1_TRADD] → [TNFR1] + [TRADD]**	Dissociation	k152	0.1	s^{-1}	(18)
**[TNFR1_TRADD] + [TAK1] → TAK1_P**	TAK1 Phosphorylation by	k153	0.1	$nM^{-1}s^{-1}$	(18)

	TNFR1_TRADD				
**[TNFR1_TRADD] → sink**	Complex Degradation	k153b	0.1	s^{-1}	(18)
**[NFkBn]^2 + [AP1]^2 + [IRF1n] + [IRF2n] + [STAT1n_P_STAT1n_P] → [iNOS_mRNAn]**	iNOS Gene Expression	k154<br>k155<br>KiNOS1<br>KiNOS2<br>KIirf2	1e-3<br>0.02<br>0.022<br>0.017<br>364	nM/s<br>nM/s<br>nM<br>nM<br>nM	(11, 13, 16, 19, 20)
**[iNOS_mRNAn] → [iNOS_mRNAc]**	Cytoplasmic transport of mRNA	k156	1e-3	s^{-1}	(1)
**[iNOS_mRNAc] → [iNOS]**	Translation	k157	0.01	s^{-1}	(1)
**[iNOS] + [arg] → [NO] + [citrulline]**	Enzymatic formation of NO	k158<br>k159	5.83e-3<br>2800	nM/s<br>nM	(5)
**[citrulline] → [arginosuccinate]**	Enzymatic formation of arginosuccinate	k160<br>k161	86<br>4.4e4	nM/s<br>nM	(5)
**[arginosuccinate] → [arg]**	Enzymatic formation of arginine	k162<br>k163	171.67<br>2.0e5	nM/s<br>nM	(5)
**[NFkBn]^2 → [IkBa_mRNAn]**	IkBa Gene Expression	k164	1.47e-2	$nM^{-1}s^{-1}$	(10)
**[IkBa_mRNAn] → [IkBa_mRNAc]**	Cytoplasmic transport of mRNA	k165	1e-3	s^{-1}	(1)
**[IkBa_mRNAc] → [IkBa]**	Translation	k166	4.08e-3	s^{-1}	(1)
**[IRF1n] → [IRF2_mRNAn]**	IRF2 Gene Expression	k170<br>k171	0.01<br>400	nM/s<br>nM	(17)
**[IRF2_mRNAn] → [IRF2_mRNAc]**	Cytoplasmic transport of mRNA	k172	1e-3	s^{-1}	(1)
**[IRF2_mRNAc] → [IRF2c]**	Translation	k173	0.01	s^{-1}	(1)
**[IRF2c] → [IRF2n]**	Nuclear Transport	k174	4.60e-3	s^{-1}	(17)

All reactions used within the model and their relative parameters can be found in supplementary table 1. The parameter values in the table above represent optimized parameters, however, they are similar to the preliminary parameters used prior to optimization. Therefore, the references provided give values of parameters prior to optimization. Interestingly, the unoptimized parameters were at most, plus or minus an order of magnitude from the optimized parameters. This can be seen visually in the graph of control iNOS expression under LPS activation before and after optimization as shown in supplementary figure 1.

Supplementary Figure 1: iNOS Gene Expression Optimization

The experimental results published by Mustafa et al were normalized and plotted against the simulated control expression of iNOS mRNA under LPS stimulation (23). **The parameters were then optimized using freely available software, DAKOTA, which was created by Sandia National Labs. Sets 1, 2, and 3 represent the three best-fitted set of parameters to the experimental results.**

The demonstrate the validity of our model, supplementary figure 2-4 show the time course dynamics of key intermediates within the iNOS expression pathway simulated under LPS and IFN-y stimulation after 24 hr priming simulation. We believe that IFN-y priming is essential to the outcome of infection and the ability of IFN-y to cause a delayed proinflammatory response is key to the outcome of infection since certain bacteria are able to evade the initial oxidative burst caused by TNF-a.

The upper and lower MAPK pathways are plotted in supplementary figure 2 and they are expressed in arbitrary units relative to control expression under LPS simulation. Interestingly, the upper MAPK pathway, that is all intermediates from LPS activated complex to ceremide production all peak near 1 however, the lower MAPK pathway composed of TAK1, SEK1, and JNK peak at higher magnitudes. This outcome supports the mechanism of IFN-y priming as it has the ability to increase large concentrations of TNF-a which can activate the lower MAPK pathway through an autocrine mechanism thus by passing the upper MAPK pathway. Furthermore, as opposed to TAK1 and SEK1, dually phosphorylated JNK is short lived since it has a higher tendency to translocate within the nucleus to form the iNOS transcription factor, AP1.

Supplementary Figure 2: MAPK Intermediates

The MAPK intermediates plotted here represent the activation propagation from LPS activated complex to upper and lower MAPK pathways and their regulatory phosphatases, MKP1 and MKP5. IFN-y priming condition was simulated for 24 hours upon which the end values of the priming were used as initial conditions for LPS and IFN-y activation condition that was simulated for 8 hours.

Supplementary Figure 3: NFkB Pathway Intermediates
The NFkB intermediates plotted here represent the activation propagation IKK to the dissociation of the IkBa-NFkB cytoplasmic complex and eventual IkBa-mRNAn expression. The rate equations and parameters were initially taken from the model published by Sharp et al (10) and the units were modified accordingly to fit our model.

The NFkB pathway intermediates are plotted in supplementary figure 3. The pathway is composed from the activation of IKK to dissociation of the IkBa-NFkBc complex forming free NFkBc. Although IKK concentrations decrease gradually, phosphorylated IKK concentrations do not see an increase until 3 hours of simulation. This is due to the usage in rapid binding of the IkBa-NFkB complex and the exponential decrease in the IkBa_NFkBc binary complex.

Supplementary Figure 4: Arginine-Citrulline Cycle
The three species plotted here represent the simulation of the arginine-citrulline cycle. Through the action of arginosuccinate synthase and arginosuccinate lyase, arginine is replenished back into the system after it's utilization by iNOS to produce NO.

The arginine-citrulline cycle plotted in supplementary figure 4 shows a cyclical expression dynamic of arginine returning back to its initial condition. The initial value of arginine was set to saturating levels to understand the mechanism of iNOS, however, the intermediates, citrulline and arginosuccinate both increase in a time dependent fashion as arginine is utilized. Furthermore, the decrease in arginosuccinate is inversely correlated to the increase in arginine back into the system by the enzymatic action of arginosuccinate lyase.

Lastly, all initial values of all dynamic species within this model under their respective stimulation have been consolidated in supplementary table 2. Whilst most species are set to zero or have a single initial condition, few species have multiple initial conditions. These values correlate to different conditions used to simulate the model under different stimulations. IFN was 0 under LPS activation only whereas it was set to 10 under

activation conditions. 1 nM IFN was used during IFN priming. The IFN receptors, R and JAK, are also set to similar values as IFN to ensure direct activation.

LPS was set to 308 nM, which if converted to density, equals 10 ng/mL, a value used in a various *in vitro* experiments (21, 22). We assumed that the LPS binding protein, LBP, would be at a similar concentration as LPS and the LPS receptors, namely, CD14, TLR4, and MD2, to be approximately one-hundredth the concentration of LPS. Finally, TNFalphaEC concentrations varied based on activation and/or priming with TNFalpha within the model. 0.5 nM was an activating condition and 0.05 was used for priming.

Species	Initial Condition (nM)
**IFN**	0, 1, 10
**R**	0, 1, 10
**JAK**	0, 1, 10
**RJ**	0
**IFNRJ**	0
**IFNRJ2**	0
**IFNRJ2_P**	0
**STAT1c**	1000
**STAT1c_P**	0
**IFNRJ2_P_STAT1c**	0
**IFNRJ2_P_STAT1c_P**	0
**STAT1c_P_STAT1c_P**	0
**SHP2**	100
**IFNRJ2_P_SHP2**	0
**PPX**	50
**PPX_STAT1c_P**	0
**PPX_STAT1c_P_STAT1c_P**	0
**STAT1c_STAT1c_P**	0
**STAT1n_P_STAT1n_P**	0
**STAT1n_P**	0

**PPN**	60
**PPN_STAT1n_P**	0
**PPN_STAT1n_P_STAT1n_P**	0
**STAT1n_STAT1n_P**	0
**STAT1n**	0
**SOCS1_mRNAn**	0
**SOCS1_mRNAc**	0
**SOCS1**	0
**IFNRJ2_P_SOCS1**	0
**IFNRJ2_P_SOCS1_STAT1c**	0
**IFNRJ2_P_SOCS1_STAT1c_SHP2**	0
**IFNRJ2_P_STAT1c_SHP2**	0
**IFNRJ2_P_SOCS1_SHP2**	0
**IFNR**	0
**LPS**	0, 308
**CD14**	(LPS/100)
**LBP**	0, 308
**MD2**	(LPS/100)
**LPS_CD14**	0
**LPS_LBP**	0
**TLR4_MD2**	0
**TLR4**	(LPS/100)
**AC1**	0
**AC2**	0
**PI3K**	10
**AC_PI3K**	0
**PI3K_P**	0
**PDK1**	10
**PI3K_P_PDK1**	0
**PDK1_P**	0

**PKC**	10
**PDK1_P_PKC**	0
**PKC_P**	0
**PCPLC**	10
**PKC_P_PCPLC**	0
**PCPLC_P**	0
**Asmase**	10
**PCPLC_P_Asmase**	0
**Asmase_star**	0
**Sphingomyelin**	100
**Ceremide**	0
**TAK1**	10
**Ceremide_TAK1**	0
**TAK1_P**	0
**SEK1**	10
**TAK1_P_SEK1**	0
**SEK1_P**	0
**TAK1_P_SEK1_P**	0
**SEK1_PP**	0
**JNK**	10
**SEK1_PP_JNK**	0
**JNK_P**	0
**SEK1_PP_JNK_P**	0
**JNK_PP**	0
**AP1**	0
**MKP1**	50
**JNK_P_MKP1**	0
**JNK_PP_MKP1**	0
**MKP5**	0
**JNK_P_MKP5**	0

**JNK_PP_MKP5**	0
**IKK**	10
**TAK1_P_IKK**	0
**IKK_P**	0
**IkBa**	0
**NFkBc**	0
**IkBa_NFkBc**	100
**IKK_P_IkBa_NFkBc**	0
**NFkBn**	0
**IkBa_mRNAn**	0
**IkBa_mRNAc**	0
**IRF1_mRNAn**	0
**IRF1_mRNAc**	0
**IRF1n**	0
**IRF1c**	0
**TNFalpha_mRNAn**	0
**TNFalpha_mRNAc**	0
**TNFalphac**	0
**TNFalphaEC**	0, 0.05, 0.5
**TR1**	308
**TNFR1**	0
**TNFR1i_TRADD**	0
**TRADD**	308
**iNOS_mRNAn**	0
**iNOS_mRNAc**	0
**iNOS**	0
**Arg**	1000
**Citrulline**	0
**Arginosuccinate**	0
**NO**	0

**IRF2_mRNAn**	0
**IRF2_mRNAc**	0
**IRF2c**	0
**IRF2n**	0

References

1. Yamada S, Shiono S, Joo A, Yoshimura A. Control mechanism of JAK/STAT signal transduction pathway. FEBS Lett. 2003 Jan 16;534(1-3):190-6.

2. Shin HJ, Lee H, Park JD, Hyun HC, Sohn HO, Lee DW, et al. Kinetics of binding of LPS to recombinant CD14, TLR4, and MD-2 proteins. Mol Cells. 2007 Aug 31;24(1):119-24.

3. Nyman E, Brannmark C, Palmer R, Brugard J, Nystrom FH, Stralfors P, et al. A hierarchical whole-body modeling approach elucidates the link between in Vitro insulin signaling and in Vivo glucose homeostasis. J Biol Chem. 2011 Jul 22;286(29):26028-41.

4. Cooper CL, Morris AJ, Harden TK. Guanine nucleotide-sensitive interaction of a radiolabeled agonist with a phospholipase C-linked P2y-purinergic receptor. J Biol Chem. 1989 Apr 15;264(11):6202-6.

5. Chang A, Schomburg I, Placzek S, Jeske L, Ulbrich M, Xiao M, et al. BRENDA in 2015: exciting developments in its 25th year of existence. Nucleic Acids Res. 2015 Jan 28;43(Database issue):D439-46.

6. Westwick JK, Bielawska AE, Dbaibo G, Hannun YA, Brenner DA. Ceramide activates the stress-activated protein kinases. J Biol Chem. 1995 Sep 29;270(39):22689-92.

7. Gutierrez J, Laurent GSI, Urcuqui-Inchima S. Propagation of kinetic uncertainties through a canonical topology of the TLR4 signaling network in dierent regions of biochemical reaction space.

Additional file 1 - Mathematical structure of the signal transductionnetwork: kinetic parameters, initial conditions, and rate equations. Theoretical Biology and Medical Modeling. 2010.

8. Dong Z, Ma W, Huang C, Yang CS. Inhibition of tumor promoter-induced activator protein 1 activation and cell transformation by tea polyphenols, (-)-epigallocatechin gallate, and theaflavins. Cancer Res. 1997 Oct 1;57(19):4414-9.

9. Liu Y, Shepherd EG, Nelin LD. MAPK phosphatases--regulating the immune response. Nat Rev Immunol. 2007 Mar;7(3):202-12.

10. Sharp GC, Ma H, Saunders PTK, Norman JE. A Computational Model of Lipopolysaccharide-Induced Nuclear Factor Kappa B Activation: A Key Signalling Pathway in Infection-Induced Preterm Labour. PLoS ONE. 2013;8(7).

11. Bergqvist S, Alverdi V, Mengel B, Hoffmann A, Ghosh G, Komives EA. Kinetic enhancement of NF-kappaBxDNA dissociation by IkappaBalpha. Proc Natl Acad Sci U S A. 2009 Nov 17;106(46):19328-33.

12. Ohmori Y, Hamilton TA. Requirement for STAT1 in LPS-induced gene expression in macrophages. J Leukoc Biol. 2001 Apr;69(4):598-604.

13. Kwon H, Park S, Lee S, Lee DK, Yang CH. Determination of binding constant of transcription factor AP-1 and DNA. Application of inhibitors. Eur J Biochem. 2001 Feb;268(3):565-72.

14. Renier G, Skamene E, DeSanctis JB, Radzioch D. Induction of tumor necrosis factor alpha gene expression by lipoprotein lipase. J Lipid Res. 1994 Feb;35(2):271-8.

15. Vila-del Sol V, Punzon C, Fresno M. IFN-gamma-induced TNF-alpha expression is regulated by interferon regulatory factors 1 and 8 in mouse macrophages. J Immunol. 2008 Oct 1;181(7):4461-70.

16. Martin E, Nathan C, Xie QW. Role of interferon regulatory factor 1 in induction of nitric oxide synthase. J Exp Med. 1994 Sep 1;180(3):977-84.

17. Coccia EM, Del Russo N, Stellacci E, Orsatti R, Benedetti E, Marziali G, et al. Activation and repression of the 2-5A synthetase and p21 gene promoters by IRF-1 and IRF-2. Oncogene. 1999 Mar 25;18(12):2129-37.

18. Grell M, Wajant H, Zimmermann G, Scheurich P. The type 1 receptor (CD120a) is the high-affinity receptor for soluble tumor necrosis factor. Proc Natl Acad Sci U S A. 1998 Jan 20;95(2):570-5.

19. Yamada S, Shiono S, Joo A, Yoshimura A. Control mechanism of JAK/STAT signal transduction pathway. FEBS Lett. 2003 Jan 16;534(1-3):190-6.

20. Wang Z, Zhang K, Wooley KL, Taylor JS. Imaging mRNA Expression in Live Cells via PNA.DNA Strand Displacement-Activated Probes. J Nucleic Acids. 2012;2012:962652.

21. Akashi S, Saitoh S, Wakabayashi Y, Kikuchi T, Takamura N, Nagai Y, et al. Lipopolysaccharide interaction with cell surface Toll-like receptor 4-MD-2: higher affinity than that with MD-2 or CD14. J Exp Med. 2003 Oct 6;198(7):1035-42.

22. Chan ED, Riches DW. IFN-gamma + LPS induction of iNOS is modulated by ERK, JNK/SAPK, and p38(mapk) in a mouse macrophage cell line. Am J Physiol Cell Physiol. 2001 Mar;280(3):C441-50.

23. Mustafa SB, Olson MS. Expression of nitric-oxide synthase in rat Kupffer cells is regulated by cAMP. J Biol Chem. 1998 Feb 27;273(9):5073-80.
