# Supplementary material for: Investigating the Role of TNF-α and IFN-γ Activation on the Dynamics of iNOS Gene Expression in LPS Stimulated Macrophages
Source: PLoS One. 2016 Jun 8;11(6):e0153289. doi: 10.1371/journal.pone.0153289 (PMC4898755; doi:10.1371/journal.pone.0153289)
Supplement: S1 Table — (PDF) [file pone.0153289.s006.pdf]

Table S1: Model Reactions and Parameters

Reaction	Function	Rate Constant	Parameters	Units	References
**[LPS]+[LBP] → [LPS-LBP]**	Binding	k77_1	2.5	$nM^{-1}s^{-1}$	(2)
**[LPS-LBP] → [LPS] + [LBP]**	Dissociation	k77_1minus	0.0251	s^{-1}	(2)
**[LPS]+[CD14] → [LPS-CD14]**	Receptor binding	k77_2	2.86e-6	$nM^{-1}s^{-1}$	(2)
**[LPS-CD14] → [LPS] + [CD14]**	Dissociation	k77_2minus	0.0251	s^{-1}	(2)
**[LPS-LBP] + [CD14] → [LPS-CD14] + [LBP]**	LPS Transfer	k77_3	0.00286	$nM^{-1}s^{-1}$	(2)
**[LPS-CD14] → [LPS] + [CD14]**	Dissociation	k77_3minus	0.0251	s^{-1}	(2)
**[TLR4]+[MD2] → [TLR4-MD2]**	Helper protein binding	k77_4	6.47e-6	$nM^{-1}s^{-1}$	(2)
**[TLR4-MD2] → [TLR4] + [MD2]**	Dissociation	k77_4minus	3.65e-4	s^{-1}	(2)
**[LPS-CD14]+[TLR4] → [AC1]**	Membrane complex 1 formation	k77_5	2.33e-6	$nM^{-1}s^{-1}$	(2)
**[AC1] → [LPS-CD14]+[TLR4]**	Dissociation	k77_5minus	6.34e-2	s^{-1}	(2)
**[LPS-CD14]+[TLR4-MD2] → [AC2]**	Membrane complex 2 formation	k77_6	4.37e-4	$nM^{-1}s^{-1}$	(2)
**[AC2] → [LPS-CD14]+[TLR4-MD2]**	Dissociation	k77_6minus	4.03e-2	s^{-1}	(2)
**[AC1]+[PI3K] → [AC-PI3K]**	Binding Reaction	k79	4.80e-4	$nM^{-1}s^{-1}$	(3)
**[AC2]+[PI3K] → [AC-PI3K]**	Binding Reaction	k79	4.80e-4	$nM^{-1}s^{-1}$	(3)
**[AC-PI3K] → sink**	Complex Degradation	k80	2.81e-5	s^{-1}	(3)
**[AC-PI3K] → [PI3K_P]**	PI3K Phosphorylation	k81	2.82e-4	s^{-1}	(3)
**[PI3K_P]+[PDK1] → [PI3K-PDK1]**	Binding Reaction	k82	7.56e-5	$nM^{-1}s^{-1}$	(3)
**[PI3K_P-PDK1] → sink**	Complex Degradation	k83	1.05e-5	s^{-1}	(3)
**[PI3K_P-PDK1] → [PDK1_P]**	PDK1 Phosphorylation	k84	6.84e-4	s^{-1}	(3)
**[PDK1_P]+[PKC] → [PDK1_P-PKC]**	Binding Reaction	k85	0.016	$nM^{-1}s^{-1}$	(3)
**[PDK1_P-PKC] → sink**	Complex Degradation	k86	7.80e-8	s^{-1}	(3)
**[PDK1_P-PKC] → [PKC_P]**	PKC Phosphorylation	k87	0.016	s^{-1}	(3)
**[PKC_P]+[PCPLC] → [PKC_P-PCPLC]**	Binding Reaction	k88	2.08e-4	$nM^{-1}s^{-1}$	(4)
**[PKC_P-PCPLC] → sink**	Complex Degradation	k89	6.33e-4	s^{-1}	(4)
**[PKC_P-PCPLC] → [PCPLC_P]**	PCPLC_P Phosphorylation	k90	0.009	s^{-1}	(4)
**[PCPLC_P]+[Asmase] → [PCPLC_P-Asmase]**	Binding Reaction	k91	1.83e-4	$nM^{-1}s^{-1}$	(4)
**[PCPLC_P-Asmase] → sink**	Complex Degradation	k92	6.33e-4	s^{-1}	(4)
**[PCPLC_P-Asmase] → [Asmase*]**	Asmase Activation	k93	0.009	s^{-1}	(4)
**[Asmase*] + [sphingomyelin] → [ceremide]**	Enzymatic Reaction	k94<br>k95	103.83<br>590	nM/s<br>nM	(5)<br>(5)

**[ceremide]+[TAK1] → [ceremide-TAK1]**	Binding Reaction	k96	3.30e-4	$nM^{-1}s^{-1}$	(6)
**[ceremide-TAK1] → sink**	Complex Degradation	k97	2.17e-3	s^{-1}	(6)
**[ceremide-TAK1] ➔ [TAK1_P]**	TAK1 Phosphorylation	k98	7.40e-3	s^{-1}	(6)
**[TAK1_P]+[SEK1] → [TAK1_P-SEK1]**	Binding Reaction	k99	2.57e-4	$nM^{-1}s^{-1}$	(7)
**[TAK1_P-SEK1] → sink**	Complex Degradation	k100	1.60e-5	s^{-1}	(7)
**[TAK1_P-SEK1] ➔ [SEK1_P]**	SEK1 Phosphorylation	k101	5.64e-3	s^{-1}	(7)
**[TAK1_P]+[SEK1_P] → [TAK1_P-SEK1_P]**	Binding Reaction	k102	2.70e-3	$nM^{-1}s^{-1}$	(7)
**[TAK1_P-SEK1_P] → sink**	Complex Degradation	k103	1.60e-5	s^{-1}	(7)
**[TAK1_P-SEK1_P] ➔ [SEK1_PP]**	SEK1_P Phosphorylation	k104	0.05	s^{-1}	(7)
**[SEK1_PP]+[JNK] → [SEK1_PP-JNK]**	Binding Reaction	k105	1.30e-3	$nM^{-1}s^{-1}$	(7)
**[SEK1_PP-JNK] → sink**	Complex Degradation	k106	1.60e-3	s^{-1}	(7)
**[SEK1_PP-JNK] ➔ [JNK_P]**	JNK Phosphorylation	k107	0.05	s^{-1}	(7)
**[SEK1_PP]+[JNK_P] → [SEK1_PP-JNK_P]**	Binding Reaction	k108	1.30e-2	$nM^{-1}s^{-1}$	(7)
**[SEK1_PP-JNK_P] → sink**	Complex Degradation	k109	1.60e-3	s^{-1}	(7)
**[SEK1_PP-JNK_P] ➔ [JNK_PP]**	JNK_P Phosphorylation	k110	0.05	s^{-1}	(7)
**[JNK_PP] ➔ [AP1]**	JNK_PP Nuclear Translocation	k111	3.40e-3	s^{-1}	(8)
**[AP1] ➔ [JNK_PP]**	AP1 Cytoplasmic Translocation	k167	1.60e-4	s^{-1}	(8)
**[JNK_P]+[MKP1] → [JNK_P-MKP1]**	Binding Reaction	k112	0.01	$nM^{-1}s^{-1}$	(9)
**[JNK_P-MKP1] → sink**	Complex Degradation	k113	1	s^{-1}	(9)
**[JNK_P-MKP1] ➔ [JNK]**	JNK_P Dephosphorylation	k114	0.05	s^{-1}	(9)
**[JNK_PP]+[MKP1] → [JNK_PP-MKP1]**	Binding Reaction	k115	0.045	$nM^{-1}s^{-1}$	(9)
**[JNK_PP-MKP1] → sink**	Complex Degradation	k116	1	s^{-1}	(9)
**[JNK_PP-MKP1] ➔ [JNK_P]**	JNK_PP Dephosphorylation	k117	0.092	s^{-1}	(9)
**[JNK_P]+[MKP5] → [JNK_P-MKP5]**	Binding Reaction	k118	0.011	$nM^{-1}s^{-1}$	(9)
**[JNK_P-MKP5] → sink**	Complex Degradation	k119	0.99	s^{-1}	(9)
**[JNK_P-MKP5] ➔ [JNK]**	JNK_P	k120	0.055	s^{-1}	(9)

	Dephosphorylation				
**[JNK_PP]+[MKP5] → [JNK_PP-MKP5]**	Binding Reaction	k121	0.046	$nM^{-1}s^{-1}$	(9)
**[JNK_PP-MKP5] → sink**	Complex Degradation	k122	0.99	s^{-1}	(9)
**[JNK_PP-MKP5] → [JNK_P]**	JNK_PP Dephosphorylation	k123	0.093	s^{-1}	(9)
**[TAK1_P]+[IKK] → [TAK1_P-IKK]**	Binding Reaction	k124	8.93e-5	$nM^{-1}s^{-1}$	(10)
**[TAK1_P-IKK] → sink**	Complex Degradation	k125	1.0e-4	s^{-1}	(10)
**[TAK1_P-IKK] → [IKK_P]**	IKK Phosphorylation	k126	0.1	s^{-1}	(10)
**[IkBa]+[NFkBc] → [IkBa_NFkBc]**	NFkBc Deactivation	k127	0.5	$nM^{-1}s^{-1}$	(10)
**[IkBa_NFkBc] → sink**	Complex Degradation	k128	3.96e-4	s^{-1}	(10)
**[IKK_P]+[IkBa_NFkBc]→[IKK_P-IkBa-NFkBc]**	Binding Reaction	k129	0.185	$nM^{-1}s^{-1}$	(10)
**[IKK_P-IkBa_NFkBc] → sink**	Complex Degradation	k130	0.0125	s^{-1}	(10)
**[IKK_P-IkBa_NFkBc] → [IKK_P] + [NFkBc]**	NFkBc Activation	k131	0.0204	s^{-1}	(10)
**[IkBa_NFkBc] → [IkBa] + [NFkBc]**	Dissociation Reaction	k132	2.61e-5	s^{-1}	(10)
**[NFkBc] → [NFkBn]**	Nuclear transport of NFkB	k133	0.09	s^{-1}	(10)
**[NFkBn] → [NFkBc]**	Cytoplasmic transport of NFkB	k134	8.0e-5	s^{-1}	(10)
**$[NFkB]^2$ + [STAT1n_P_STAT1n_P] → [IRF1_mRNAn]**	IRF1 Gene Expression	k135<br>k135b<br>k136<br>k137<br>KIirf2	4.43e-3<br>0.03<br>1.94<br>10<br>364	nM/s<br>nM/s<br>nM<br>nM<br>nM	(1, 11, 12)
**[IRF1_mRNAn] → [IRF1_mRNAc]**	Cytoplasmic transport of mRNA	k138	1.36e-3	s^{-1}	(1)
**[IRF1_mRNAc] → [IRF1c]**	Translation	k139	0.01	s^{-1}	(1)
**[IRF1c] → [IRF1n]**	Nuclear Transport of IRF1	k140	5e-3	s^{-1}	(1)
**$[NFkB]^3$ + [AP1] → [TNFalpha_mRNAn]**	TNF-a Gene Expression	k141<br>k142<br>k143	0.01<br>3<br>4	nM/s<br>nM<br>nM	(11, 13, 14)
**$[IRF1n]^2$ + [IRF2n]→ [TNFalpha_mRNAn]**	TNF-a Gene Expression	k168<br>k169<br>KIirf2	1.38e-3<br>4.99e-3<br>364	nM/s<br>nM<br>nM	(15-17)
**[TNFalpha_mRNAn] → [TNFalpha_mRNAc]**	Cytoplasmic transport of mRNA	k144	8.05e-4	s^{-1}	(1)
**[TNFalpha_mRNAc] → [TNFalphac]**	Translation	k145	0.01	s^{-1}	(1)
**[TNFalphac] → [TNFalphaEC]**	Cellular Export of TNFa	k146	0.1	s^{-1}	(1)
**[TNFalphaEC] + [TR1] → [TNFR1]**	Receptor Binding	k147	0.0183	$nM^{-1}s^{-1}$	(18)
**[TNFR1] → [TNFalphaEC] + [TR1]**	Dissociation	k148	3.5e-4	s^{-1}	(18)
**[TNFR1] + [TRADD] ←→ [TNFR1_TRADD]**	Membrane complex formation	k151	0.1	$nM^{-1}s^{-1}$	(18)
**[TNFR1_TRADD] → [TNFR1] +**	Dissociation	k152	0.1	s^{-1}	(18)

**[TRADD]**					
**[TNFR1_TRADD] + [TAK1] → TAK1_P**	TAK1 Phosphorylation by TNFR1_TRADD	k153	0.1	$nM^{-1}s^{-1}$	(18)
**[TNFR1_TRADD] → sink**	Complex Degradation	k153b	0.1	s^{-1}	(18)
**$[NFkBn]^2$ + $[AP1]^2$ + [IRF1n] + [IRF2n] + [STAT1n_P_STAT1n_P] → [iNOS_mRNAn]**	iNOS Gene Expression	k154<br>k155<br>KiNOS1<br>KiNOS2<br>KIirf2	1e-3<br>0.02<br>0.022<br>0.017<br>364	nM/s<br>nM/s<br>nM<br>nM<br>nM	(11, 13, 16, 19, 20)
**[iNOS_mRNAn] → [iNOS_mRNAc]**	Cytoplasmic transport of mRNA	k156	1e-3	s^{-1}	(1)
**[iNOS_mRNAc] → [iNOS]**	Translation	k157	0.01	s^{-1}	(1)
**[iNOS] + [arg] → [NO] + [citrulline]**	Enzymatic formation of NO	k158<br>k159	5.83e-3<br>2800	nM/s<br>nM	(5)
**[citrulline] → [arginosuccinate]**	Enzymatic formation of arginosuccinate	k160<br>k161	86<br>4.4e4	nM/s<br>nM	(5)
**[arginosuccinate] → [arg]**	Enzymatic formation of arginine	k162<br>k163	171.67<br>2.0e5	nM/s<br>nM	(5)
**$[NFkBn]^2$ → [IkBa_mRNAn]**	IkBa Gene Expression	k164	1.47e-2	$nM^{-1}s^{-1}$	(10)
**[IkBa_mRNAn] → [IkBa_mRNAc]**	Cytoplasmic transport of mRNA	k165	1e-3	s^{-1}	(1)
**[IkBa_mRNAc] → [IkBa]**	Translation	k166	4.08e-3	s^{-1}	(1)
**[IRF1n] → [IRF2_mRNAn]**	IRF2 Gene Expression	k170<br>k171	0.01<br>400	nM/s<br>nM	(17)
**[IRF2_mRNAn] → [IRF2_mRNAc]**	Cytoplasmic transport of mRNA	k172	1e-3	s^{-1}	(1)
**[IRF2_mRNAc] → [IRF2c]**	Translation	k173	0.01	s^{-1}	(1)
**[IRF2c] → [IRF2n]**	Nuclear Transport	k174	4.60e-3	s^{-1}	(17)
