# Supplementary material for: Investigating the Role of TNF-α and IFN-γ Activation on the Dynamics of iNOS Gene Expression in LPS Stimulated Macrophages
Source: PLoS One. 2016 Jun 8;11(6):e0153289. doi: 10.1371/journal.pone.0153289 (PMC4898755; doi:10.1371/journal.pone.0153289)
Supplement: S2 Table — (PDF) [file pone.0153289.s007.pdf]

Table S2: Initial Conditions of all Reaction Species within the Model

Species	Initial Condition (nM)
**IFN**	0, 1, 10
**R**	0, 1, 10
**JAK**	0, 1, 10
**RJ**	0
**IFNRJ**	0
**IFNRJ2**	0
**IFNRJ2_P**	0
**STAT1c**	1000
**STAT1c_P**	0
**IFNRJ2_P_STAT1c**	0
**IFNRJ2_P_STAT1c_P**	0
**STAT1c_P_STAT1c_P**	0
**SHP2**	100
**IFNRJ2_P_SHP2**	0
**PPX**	50
**PPX_STAT1c_P**	0
**PPX_STAT1c_P_STAT1c_P**	0
**STAT1c_STAT1c_P**	0
**STAT1n_P_STAT1n_P**	0
**STAT1n_P**	0
**PPN**	60
**PPN_STAT1n_P**	0
**PPN_STAT1n_P_STAT1n_P**	0
**STAT1n_STAT1n_P**	0
**STAT1n**	0
**SOCS1_mRNAn**	0
**SOCS1_mRNAc**	0
**SOCS1**	0
**IFNRJ2_P_SOCS1**	0
**IFNRJ2_P_SOCS1_STAT1c**	0
**IFNRJ2_P_SOCS1_STAT1c_SHP2**	0
**IFNRJ2_P_STAT1c_SHP2**	0
**IFNRJ2_P_SOCS1_SHP2**	0
**IFNR**	0
**LPS**	0, 308
**CD14**	(LPS/100)
**LBP**	0, 308
**MD2**	(LPS/100)
**LPS_CD14**	0
**LPS_LBP**	0
**TLR4_MD2**	0
**TLR4**	(LPS/100)
**AC1**	0
**AC2**	0
**PI3K**	10
**AC_PI3K**	0
**PI3K_P**	0
**PDK1**	10
**PI3K_P_PDK1**	0
**PDK1_P**	0
**PKC**	10
**PDK1_P_PKC**	0
**PKC_P**	0
**PCPLC**	10
**PKC_P_PCPLC**	0
**PCPLC_P**	0

**Asmase**	10
**PCPLC_P_Asmase**	0
**Asmase_star**	0
**Sphingomyelin**	100
**Ceremide**	0
**TAK1**	10
**Ceremide_TAK1**	0
**TAK1_P**	0
**SEK1**	10
**TAK1_P_SEK1**	0
**SEK1_P**	0
**TAK1_P_SEK1_P**	0
**SEK1_PP**	0
**JNK**	10
**SEK1_PP_JNK**	0
**JNK_P**	0
**SEK1_PP_JNK_P**	0
**JNK_PP**	0
**AP1**	0
**MKP1**	50
**JNK_P_MKP1**	0
**JNK_PP_MKP1**	0
**MKP5**	0
**JNK_P_MKP5**	0
**JNK_PP_MKP5**	0
**IKK**	10
**TAK1_P_IKK**	0
**IKK_P**	0
**IkBa**	0
**NFkBc**	0
**IkBa_NFkBc**	100
**IKK_P_IkBa_NFkBc**	0
**NFkBn**	0
**IkBa_mRNAn**	0
**IkBa_mRNAc**	0
**IRF1_mRNAn**	0
**IRF1_mRNAc**	0
**IRF1n**	0
**IRF1c**	0
**TNFalpha_mRNAn**	0
**TNFalpha_mRNAc**	0
**TNFalphac**	0
**TNFalphaEC**	0, 0.05, 0.5
**TR1**	308
**TNFR1**	0
**TNFR1i_TRADD**	0
**TRADD**	308
**iNOS_mRNAn**	0
**iNOS_mRNAc**	0
**iNOS**	0
**Arg**	1000
**Citrulline**	0
**Arginosuccinate**	0
**NO**	0
**IRF2_mRNAn**	0
**IRF2_mRNAc**	0
**IRF2c**	0
**IRF2n**	0
